# *AoMbp1* Governs Conidiation and Trap Morphogenesis in *Arthrobotrys oligospora* Via Direct Transcriptional Activation of the MAPK Sensor *AoSho1*

**DOI:** 10.3390/jof11100736

**Published:** 2025-10-13

**Authors:** Ruobing Li, Lixiang Wei, Yanseng Sun, Chengzhi Zhang, Yuhang Nie, Qinglong Meng, Shuang Chen, Ming Wu, Xuepeng Cai, Jie Li, Qingling Meng, Jun Qiao

**Affiliations:** 1 College of Animal Science and Technology, Shihezi University, Shihezi 832003, China; 2 State Key Laboratory of Veterinary Etiological Biology, Lanzhou Veterinary Research Institute, Chinese Academy of Agricultural Sciences, Lanzhou 730046, China

**Keywords:** *Arthrobotrys oligospora*, *AoMbp1*, *AoSho1*, MAPK signaling pathway, trap formation

## Abstract

The nematode-trapping fungus (NTF) *Arthrobotrys oligospora* (*A. oligospora*) is a promising biocontrol agent, but the transcriptional regulators governing its predation remain poorly understood. Here, we demonstrated that the APSES transcription factor *AoMbp1* is a master regulator of its development and stress adaptation. Deletion of *AoMbp1* severely impaired mycelial growth, conidiation, trap formation, and tolerance to oxidative and osmotic stresses. Transcriptome analysis revealed that these defects were associated with the widespread downregulation of genes, including those within the MAPK signaling pathway. Crucially, we showed that *AoMbp1* directly binds to the promoter of *AoSho1*, a key upstream sensor of the MAPK cascade, and activates its expression. This finding establishes a direct *AoMbp1*-*AoSho1* regulatory axis controlling trap morphogenesis and environmental adaptation. Our study provides novel mechanistic insights into the regulation of nematode trapping and identifies a potential target for enhancing the efficacy of *A. oligospora* as a biocontrol agent.

## 1. Introduction

Gastrointestinal nematodes (GINs) are widespread parasites in herbivorous species, posing a substantial challenge to the sustainable development of the global herbivore industry [1]. Infections caused by nematodes from genera such as *Ostertagia*, *Trichostrongylus*, *Haemonchus* and *Nematodirus* have been shown to result in increased feed costs, growth retardation, weakened immunity, and even mortality in young herbivores [2,3]. These consequences lead to significant economic losses in the herbivore industry [4].

Currently, the application of chemical dewormers during the breeding process is a common strategy for preventing and managing gastrointestinal nematode infections in livestock. However, the widespread use of these chemical treatments has led to severe challenges, including the development of drug-resistant worm strains, the accumulation of drug residues in animal products, and environmental contamination [5]. Therefore, there is an urgent need to develop innovative methods for the prevention and control of gastrointestinal nematode diseases in herbivores to ensure the sustainable and healthy growth of the animal husbandry sector.

*Arthrobotrys oligospora* (*A. oligospora*), a filamentous fungus, has been shown to produce specialized structures such as mycorrhizal rings and adhesive mycorrhizal webs [6,7], which function as snares to capture nematode larvae [8,9]. Owing to its predatory behavior, this fungus has demonstrated significant potential in the biocontrol of nematode diseases. Nevertheless, the mechanisms governing the spore-producing capabilities and predatory activity of *A. oligospora*, one of the most promising microorganisms for biocontrol applications, remain incompletely understood. Among the transcription factors identified in filamentous fungi, the APSES family constitutes a distinct class of fungal regulatory proteins. These transcription factors feature helix-loop-helix DNA-binding domains that play key roles in essential biological processes, including fungal growth, spore production, cell differentiation, secondary metabolite biosynthesis, and pathogenicity [10,11]. In *Saccharomyces cerevisiae*, the APSES family member Mbp1 regulates the expression of genes involved in the G1/S phase transition by binding to the Mlu1 cell cycle box (MCB box) in promoter regions. Deletion of *Mbp1* has been associated with impaired pseudohyphal differentiation and disruptions in respiratory metabolism [12,13]. Similarly, Mbp1 in *Histoplasma capsulatum* influences morphological transitions between yeast and mycelial forms while also modulating pathogenicity and oxidative stress tolerance [14]. In *Pleurotus ostreatus*, Mbp1 plays a pivotal role in regulating β-glucan and chitin synthesis [15,16]. In *Beauveria bassiana*, Mbp1 is essential for mycelial differentiation and virulence expression [17,18]. Similarly, in *Fusarium verticillioides*, Mbp1 is critical for nutrient uptake, conidial development, stress response, and pathogenicity [19]. Despite substantial research into the role of Mbp1 in other fungal systems, its biological function in *A. oligospora* remains elusive. In a previous study conducted by our group, we discovered that the expression levels of *AoMbp1* significantly increase during both the spore-producing and trap-forming stages of *A. oligospora* (Figure S1). This observation suggests that *AoMbp1* is intricately linked to the development of spore-producing and trap-forming structures in this fungus.

This study aimed to bridge this knowledge gap by systematically investigating the function of *AoMbp1* in the nematode-trapping fungus *A. oligospora*, with a focus on its potential role in connecting developmental processes with stress adaptation pathways. We demonstrated that *AoMbp1* coordinates growth, conidiation, trap formation, and stress responses by directly regulating *AoSho1*, a key upstream component of the MAPK signaling pathway. This work provides new insights into the transcriptional mechanisms governing fungal predation.

## 2. Materials and Methods

### 2.1. Plasmids, Strains, Nematodes and Culture Conditions

The pCSN44 plasmid, which carries the hygromycin resistance gene (*hph*), and the pPk2-Bar plasmid, which contains the glufosinate resistance gene (*bar*), were obtained from the Key Laboratory of Preventive Veterinary Medicine at Shihezi University. The recombinant plasmids were constructed using the *E. coli* strain DH5α, which was cultured in LB media at 37 °C. The *A. oligospora* XJ-2 wild-type (WT) strain was also obtained from the same laboratory and cultured on YPSSA media at 28 °C. Additionally, *Caenorhabditis elegans* was cultured on NGM at 26 °C for 6–7 days prior to the bioassays.

### 2.2. Analysis of the Molecular Characteristics of the AoMbp1 Protein

The homologous protein AOL_s00075g215 (*AoMbp1*) was identified in the *A. oligospora* genome through BLAST analysis, and the protein sequence of *Saccharomyces cerevisiae* Mbp1 (CAA98618) was used as a query. Primers P1/P2 were designed on the basis of the *AoMbp1* gene sequence, and the gene was subsequently amplified and sequenced via PCR (Table S1). The molecular characterization of the protein encoded by the *AoMbp1* gene was conducted via DNAMAN software (https://www.lynnon.com/dnaman.html, accessed on 5 May 2025), the ExPASy online tool (https://web.expasy.org/translate/, accessed on 5 May 2025), and ScanProsite (https://prosite.expasy.org/scanprosite/, accessed on 5 May 2025). Additionally, MEGA 11 software was used to construct a phylogenetic tree of the proteins belonging to the APSES family. The nucleotide sequence of the *AoMbp1* gene has been deposited in the NCBI GenBank database under accession number PV872033 (https://www.ncbi.nlm.nih.gov/nuccore, accessed on 5 May 2025).

### 2.3. Construction of AoMbp1 Gene Deletion and Complementation Strains

The deletion strain Δ*AoMbp1* and the complementation strain CΔ*AoMbp1* were constructed through homologous recombination. Briefly, the primers P3/P4, P5/P6, and P7/P8 were specifically designed to amplify the homology arm sequences flanking the 5′ and 3′ regions of the *AoMbp1* gene, along with the PtrpC-Hph expression cassette (Table S1). Gene splicing by overlap extension PCR (SOE-PCR) was employed to construct the knockout vectors, which were subsequently transformed into protoplasts of the wild-type (WT) strain. To generate the complementation strain CΔ*AoMbp1*, the plasmid pUC19-CΔ*AoMbp1* was assembled via seamless cloning. Using this plasmid as a template, the primers PM1/PM2 were designed to amplify the full-length complement fragment (Table S1). The complement fragment was then introduced into the protoplasts of the confirmed Δ*AoMbp1* deletion strain via protoplast transformation. PCR amplification screening was performed via primers P1/P2 to identify the Δ*AoMbp1* gene deletion mutant and the CΔ*AoMbp1* complementation strain.

### 2.4. Phenotypic Analysis of Different Strains

The WT, Δ*AoMbp1*, and CΔ*AoMbp1* strains were cultured on TYGA, PDA, and TG solid media, respectively, at 28 °C for 5 days, with three biological replicates per strain. Colony morphology and aerial mycelial growth were assessed, and colony diameters were measured daily. To evaluate conidiation, germination rates, and surface hydrophobicity, the strains were incubated on CMY medium at 28 °C for 8 days in three independent experiments. Fresh conidia and hyphae from each strain were stained with calcofluor white (CFW) to observe septa in both hyphae and conidia. For trap formation assays, each strain was grown on CMA medium. Three holes were punched along the periphery of the colony, and after mycelia grew into the holes, approximately 200 nematodes were added to each well to induce trap formation. The numbers of traps and captured nematodes were recorded for each strain at 24 h and 48 h post-induction, with three biological replicates. Furthermore, each strain was incubated in PL-4 liquid medium at 28 °C for 6 days. The culture broth was then filtered, and extracellular protease activity was assessed qualitatively by measuring the size of transparent zones on skim milk powder plates in three independent experiments.

### 2.5. Analysis of Stress Tolerance

Mycelial plugs of each strain were inoculated onto TG agar plates supplemented with specific chemical stressors and incubated at 28 °C for 5 days. For hyperosmotic stress, the medium was supplemented with sodium chloride (0.1 or 0.2 mol/L) or sorbitol (0.25 or 0.5 mol/L). For oxidative stress, hydrogen peroxide (5 or 10 mmol/L) or menadione (0.03 or 0.05 mmol/L) was added to the medium. After incubation, colony diameters were measured, and the relative growth inhibition (RGI) was calculated as previously described. The ability of each strain to utilize different carbon and nitrogen sources was evaluated on minimal medium (MM) supplemented with various fatty acids (50 mM sodium acetate, 0.12% oleic acid, or 0.5% Tween 20) or nitrogen sources (urea, peptone, or yeast extract). Colony diameters were recorded after 5 days of incubation at 28 °C, and the RGI was calculated. All experiments were performed with at least three biological replicates [20,21].

### 2.6. Transcriptome Sequencing and Analysis of Differentially Expressed Genes

Briefly, the WT and Δ*AoMbp1* strains were cultured on YPSSA media at 28 °C for 6 days. Mycelial samples, each with three biological replicates, were collected and sent to Shanghai Meiji Biomedical Science and Technology Co., Ltd. (Shanghai, China), for transcriptome sequencing. The raw RNA-seq data have been deposited in the NCBI Sequence Read Archive under BioProject accession PRJNA1292889. These resulting transcriptome data were subsequently analyzed via a cloud platform. The differentially expressed genes (DEGs) were subjected to Gene Ontology (GO) functional enrichment analysis and Kyoto Encyclopedia of Genes and Genomes (KEGG) pathway enrichment analysis. Moreover, five DEGs were randomly selected, and their transcriptomic data were validated through reverse transcription–quantitative polymerase chain reaction (RT-qPCR). Additionally, potential proteins that interact with *AoMbp1* were examined via the STRING protein-protein interaction network platform. Protein-protein interaction (PPI) networks were visualized and analyzed with Cytoscape (v.3.10.0). In addition, JASPAR software (https://jaspar.elixir.no/, 5 May 2025) was used to screen and identify potential target genes regulated by *AoMbp1*.

### 2.7. Molecular Docking Analysis of AoMbp1 with the Promoter of a Downstream Target Gene

To screen candidate downstream target genes identified from transcriptome data, molecular docking was performed between *AoMbp1* and the promoter DNA. The three-dimensional structures of the *AoMbp1* protein and the promoter DNA fragment were modeled using AlphaFold 3 [22]. Protein-DNA docking was carried out with the HDOCK server. The complex with the highest confidence score was selected for further analysis of its binding interface using PDBePISA (https://www.ebi.ac.uk/pdbe/pisa/ accessed on 5 May 2025) and visualized with PyMOL, v.3. As molecular docking provides predictive insights, the top-ranked candidate interaction was subsequently validated experimentally using yeast one-hybrid (Y1H) and electrophoretic mobility shift assays (EMSA).

### 2.8. Yeast One-Hybrid (Y1H) Assay

The promoter fragment of the target gene (1043 bp) was amplified via the primers proAoSho1-F/proAoSho1-R (Table S1). It was seamlessly ligated into the pAbAi vector, generating the recombinant bait vector proAoSho1-AbAi. This recombinant vector was subsequently transformed into Y1HGold yeast strains, and the transformants were cultivated on SD/-Ura media to develop the yeast bait strain. The appropriate AbA screening concentration was then determined. Simultaneously, the full-length cDNA sequence of *AoMbp1* was amplified via the primers *AoMbp1*-F/*AoMbp1*-R (Table S1). It was ligated into the pGADT7 vector to construct the pGADT7-*AoMbp1* vector. The pGADT7-*AoMbp1* plasmid, along with the pGADT7 empty plasmid (serving as the negative control), was transformed into yeast bait strain receptor cells. The transformation products were plated on SD/-Leu/-Ura media and incubated at a constant temperature of 30 °C for 3–5 days. After that, the positive clones identified on the primary screening plates were cultured overnight in liquid SD/-Leu media, and the resulting cultures were diluted to OD_600nm_ values of 0.2, 0.02, and 0.002. These dilutions were subsequently spotted onto SD/-Leu/AbA screening media containing the same AbA concentration. The plates were subsequently sealed and incubated at 30 °C for 3–5 days, after which growth was observed.

### 2.9. Electrophoretic Mobility Shift Assay (EMSA)

Briefly, the recombinant protein *AoMbp1* was successfully produced via a prokaryotic expression system. The coding sequence of *AoMbp1* was amplified from cDNA via F1/R1 primers, and the recombinant vector pMD19-*AoMbp1* was constructed (Table S1). Both the pMD19-*AoMbp1* and pET-32a plasmids were enzymatically digested with *Kpn*I and *Hind*III, respectively. Following cleavage, the fragments of the pET-32a vector and *AoMbp1* were recovered. Under the action of T4 DNA ligase, the target fragment was ligated, resulting in the formation of the recombinant plasmid pET-*AoMbp1*, which was subsequently transformed into BL21 (DE3). The expression of the recombinant product was induced with IPTG for six hours, after which the *AoMbp1* protein was purified via a His-Ni affinity purification column. The efficiency of protein expression and purification was analyzed through SDS-PAGE and Western blot techniques. Additionally, the promoter DNA fragment of the *AoSho1* gene (1043 bp) was amplified via the primer pair F2/R2 (Table S1). The *AoSho1* promoter fragment was then incubated with varying concentrations of purified *AoMbp1* protein in gel shift buffer for 30 min at room temperature. Bovine serum albumin (BSA) was included as a nonspecific control protein. The resulting mixtures were subjected to 1% agarose gel electrophoresis for analysis, and the results were documented through imaging [23].

### 2.10. Statistical Analysis

Statistical analyses were performed using GraphPad Prism 5.0 software. Data are presented as means ± standard deviation (SD). Unpaired two-tailed Student’s *t*-tests were used for comparisons between two groups. For comparisons among multiple groups, one-way analysis of variance (ANOVA) was applied, followed by Tukey’s Post Hoc test. Differences were considered statistically significant at * *p* < 0.05, ** *p* < 0.01, and *** *p* < 0.001.

## 3. Results

### 3.1. Molecular Characterization of the Transcription Factor AoMbp1 of A. oligospora

**Figure 1:**
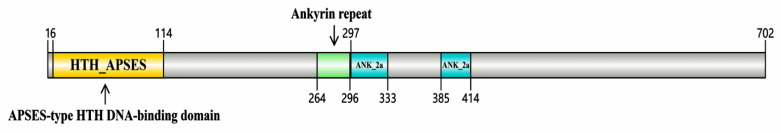
Molecular characterization of *AoMbp1*, an APSES transcription factor in the *A. oligospora* XJ-2 strain, showing its major functional motifs and domains.

Phylogenetic analysis of the APSES family of transcription factors revealed the presence of five APSES family members in the *A. oligospora* genome: *AoMbp1* (AOL_s00075g215), AoSwi6 (AOL_s00109g110), AoStuA (AOL_s00083g25), AoXbp1 (AOL_s00006g32), and AoBfp4 (AOL_s00109g183) (Figure S2). On the basis of the complete genome sequence of *A. oligospora* in NCBI, a 357 bp sequence between AOL_s00075g214 and AOL_s00075g215, which can drive the expression of these two genes and is a bidirectional promoter, was identified (Figure S3). The *AoMbp1* gene (GenBank accession: PV872033) from the *A. oligospora* XJ-2 wild-type (WT) strain comprises 2330 bp, containing three introns and an open reading frame (ORF) of 2109 bp. The ORF encodes a protein comprising 702 amino acids. Moreover, the *AoMbp1* protein includes a conserved APSES-type helix-turn-helix (HTH) DNA-binding domain along with two ankyrin repeat domains (Figure 1). Comparison of amino acid sequences revealed homology rates of 51.28%, 49.66%, and 50.59% between *AoMbp1* and the APSES domain proteins of *Tuber magnatum*, *Ascodesmis nigricans*, and *Wilcoxina mikolae*, respectively (Figure S4). Furthermore, the phylogenetic tree revealed that *AoMbp1* is highly conserved with the APSES domain proteins of *Saitoella complicata*, *Dacryopinax primogenitus*, *Fomitiporia mediterranea* MF3/22, and *Guyanagaster necrorhizus* MCA 3950 (Figure S5).

### 3.2. AoMbp1 Is Involved in the Regulation of Mycelial Growth

**Figure 2:**
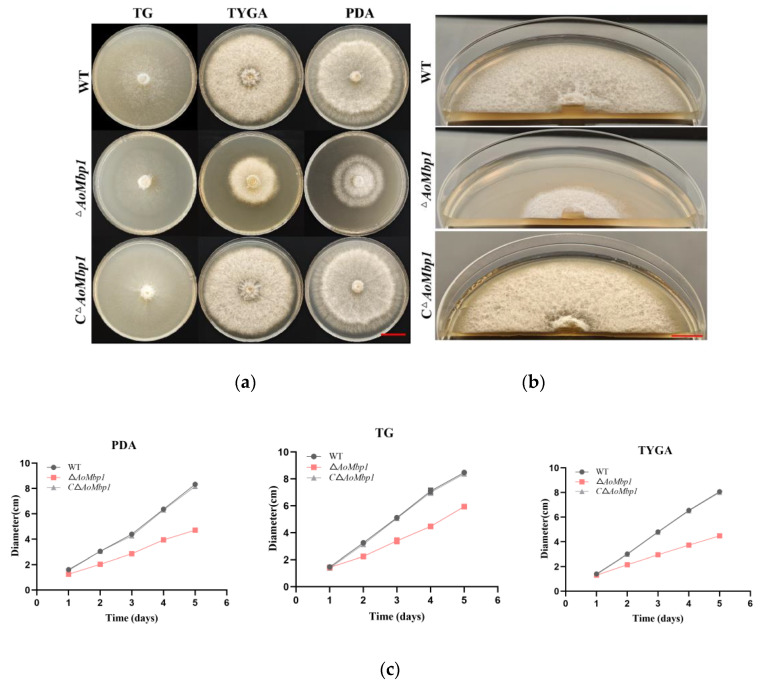

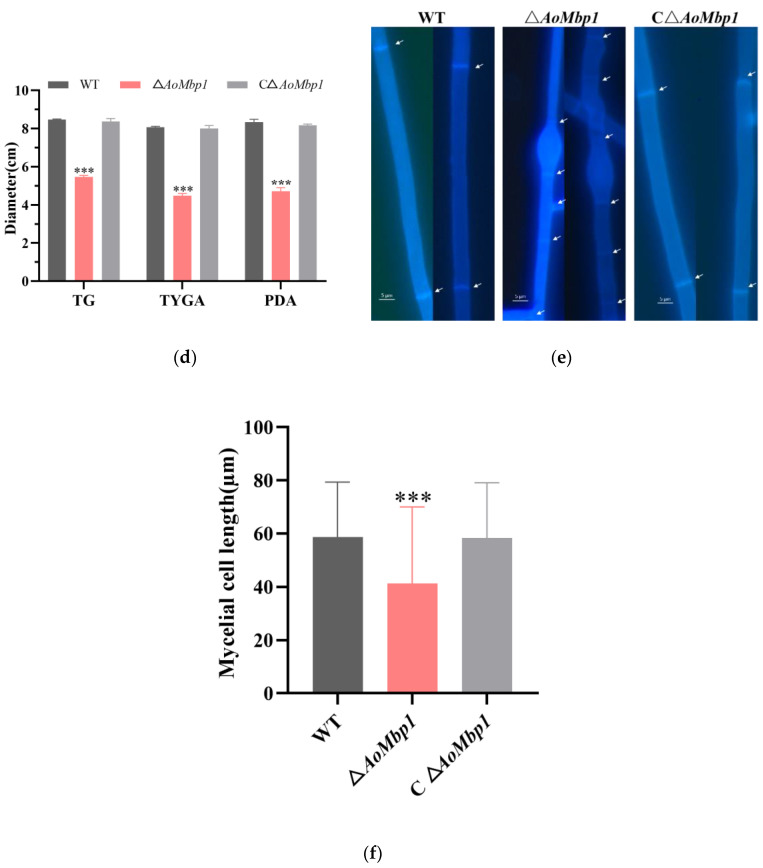
Deletion of *AoMbp1* alters hyphal growth and septation. (**a**) Colony morphology of the WT, Δ*AoMbp1*, and CΔ*AoMbp1* strains cultured on TYGA, PDA, and TG media for 5 days at 28 °C. Scale bar, 2 cm. (**b**) Cross-sections of hyphae from each strain grown on TYGA medium for 5 days. Scale bar, 1 cm. (**c**) Growth assessment of each strain on different solid media. (**d**) Colony diameters were measured on day 5. Data represent mean ± SD (*n* = 3 independent biological replicates). *** *p* < 0.001 vs. WT(one-way ANOVA with Tukey’s HSD test). (**e**) Hyphal septation visualized by CFW staining. Arrows indicate septa. Scale bar, 5 μm. (**f**) Hyphal cell length analysis. The distance between adjacent septa was measured for 125 hyphal cells per strain using ImageJ 1.49. Data are presented as mean ± SD. *** *p* < 0.001.

The Δ*AoMbp1* deletion strain and CΔ*AoMbp1* complementation strain were constructed via homologous recombination (Figure S6a–d). Compared with the WT strain, the CΔ*AoMbp1* strain did not significantly differ. In contrast, the mycelia of the Δ*AoMbp1* deletion strain presented a distinct trophic growth defect (Figure 2a), characterized by a flattened, compact colony morphology and a notable reduction in aerial mycelia (Figure 2b), relative to both the WT strain and the CΔ*AoMbp1* strain. Furthermore, the growth rates of the Δ*AoMbp1* deletion strains were significantly lower on TG, TYGA, and PDA media (Figure 2c,d). Additionally, some hyphae were swollen and presented an increased number of mycelial septa (Figure 2e), resulting in shorter mycelial cell lengths (Figure 2f).

### 3.3. Deletion of AoMbp1 Impairs Conidial Production and Morphology

**Figure 3:**
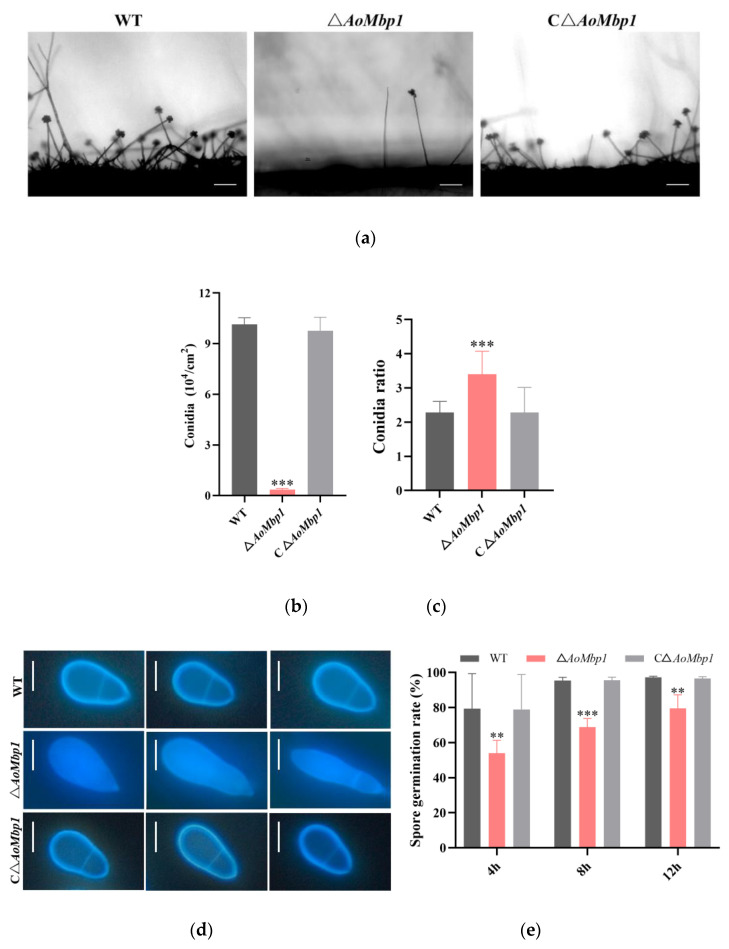

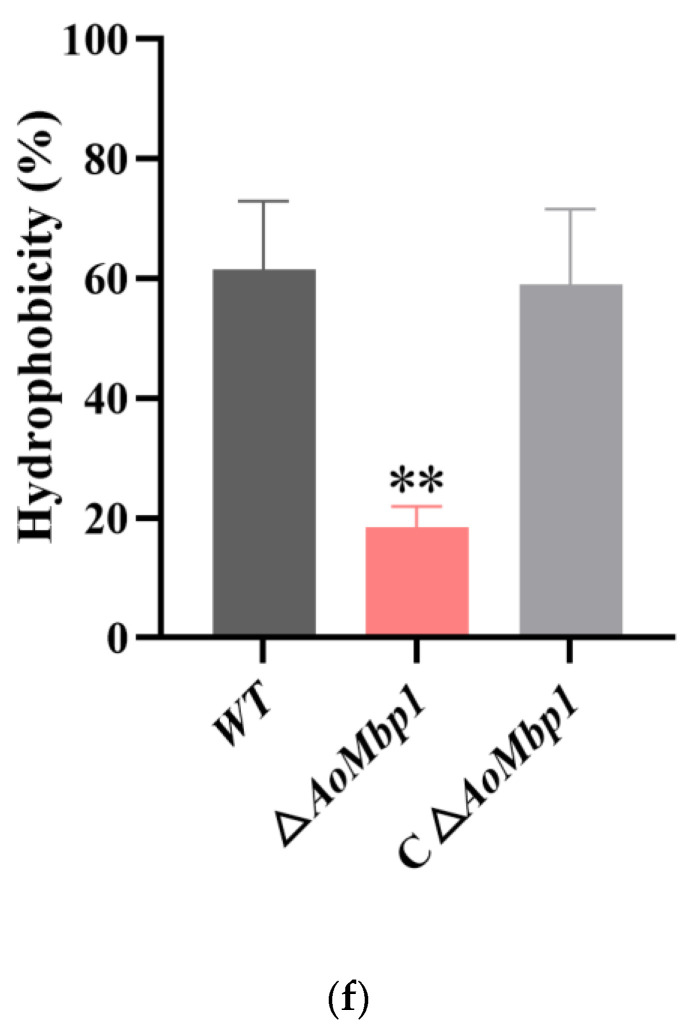
Deletion of *AoMbp1* impairs conidial production, morphology, germination, and surface hydrophobicity. (**a**) Conidial yield of each strain. Scale bar, 100 μm. (**b**) Quantification of conidial production. Data are presented as mean ± SD (*n* = 3 biological replicates). *** *p* < 0.001 (Tukey’s test). (**c**) Conidial morphology analysis based on the spore length-to-width ratio. Measurements were obtained from 60 randomly selected conidia per strain. Data are presented as mean ± SD. *** *p* < 0.001. (**d**) Calcofluor white staining revealing septation defects in Δ*AoMbp1* conidia. Scale bar, 10 μm. (**e**) Conidial germination rates. Data represent mean ± SD (*n* = 3). ** *p* < 0.01, *** *p* < 0.001. (**f**) Hydrophobicity assay of conidial surfaces. Data are shown as mean ± SD from three independent experiments. ** *p* < 0.01.

No significant differences were detected between the CΔ*AoMbp1* and WT strains. In contrast, the Δ*AoMbp1* deletion strain produced fewer conidiophores (Figure 3a) and exhibited a marked reduction in conidial yield compared to both the WT and CΔ*AoMbp1* strains (Figure 3b). Approximately 84.28% of Δ*AoMbp1* conidia displayed abnormal morphology. The length of WT conidia ranged from 18.24 to 27.55 μm (mean 21.31 μm), with a width of 7.38 to 12.07 μm (mean 9.44 μm). In comparison, Δ*AoMbp1* conidia were 15.40–63.29 μm in length (mean 33.89 μm) and 4.74–20.29 μm in width (mean 10.39 μm). The length-to-width ratio of Δ*AoMbp1* conidia was approximately 1.5 times greater than that of WT conidia, indicating an elongated and slender morphology; some conidia also exhibited incomplete septation (Figure 3c,d). The conidial germination rate of the Δ*AoMbp1* strain was significantly lower than those of the WT and CΔ*AoMbp1* strains (Figure 3e). Furthermore, the hydrophobicity of the conidial surface was significantly reduced in the mutant (Figure 3f).

### 3.4. AoMbp1 Contributes to Trap Formation, Predatory Capability and Extracellular Protein Hydrolase Expression

**Figure 4:**
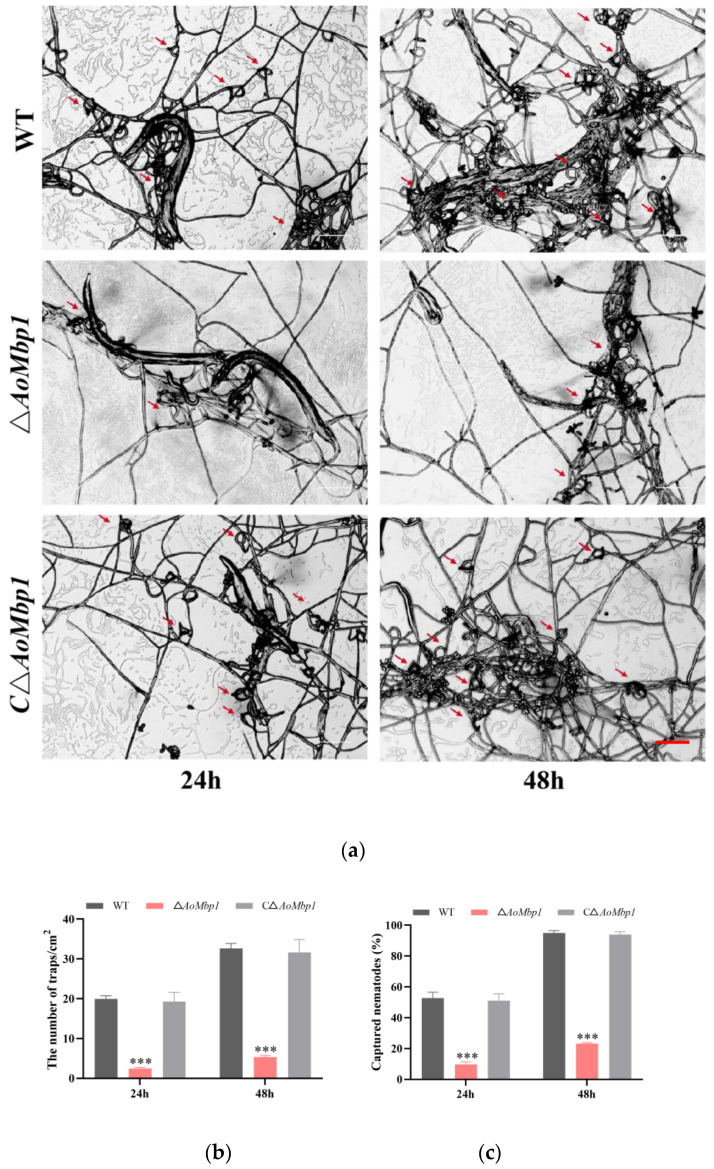

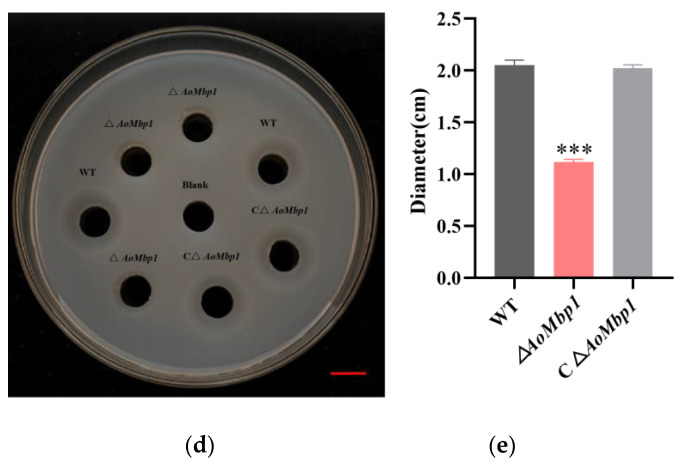
Deletion of *AoMbp1* impairs trap formation, nematode predation, and extracellular protease activity. (**a**) Representative images of predatory traps at 24 h and 48 h post-induction with nematodes. Scale bar, 200 μm. (**b**) Quantification of trap numbers. Data are presented as mean ± SD (*n* = 3 independent biological replicates). *** *p* < 0.001 (one-way ANOVA with Tukey’s test). (**c**) Nematode predation rates. Values are mean ± SD (*n* = 3). *** *p* < 0.001. (**d**) Proteolytic activity assay on skim milk agar plates. Scale bar, 1 cm. (**e**) Diameters of proteolytic zones. Data represent mean ± SD (*n* = 3). *** *p* < 0.001.

Phenotypic analysis revealed significantly reduced trap formation in the Δ*AoMbp1* strain compared to the WT and CΔ*AoMbp1* strains (Figure 4a,b). At 24 h post-nematode induction, the WT strain produced approximately 19.90 traps per cm^2^, while the Δ*AoMbp1* mutant formed only 2.48 traps per cm^2^. After 48 h, trap numbers reached 32.64 per cm^2^ in the WT strain compared to only 5.31 per cm^2^ in the mutant strain (Figure 4b). The nematode predation rate of the WT strain was 52.70% at 24 h and increased to 94.92% at 48 h, whereas the Δ*AoMbp1* strain exhibited significantly reduced predation rates of only 9.60% and 23.18%, respectively (Figure 4c). In contrast, the CΔ*AoMbp1* strain showed no significant differences from the WT strain in either trap formation or predatory capacity. Furthermore, the Δ*AoMbp1* strain displayed a significantly smaller hydrolysis zone than both the WT and CΔ*AoMbp1* strains in extracellular protease assays (Figure 4d,e), indicating that *AoMbp1* also contributes to regulating the production or secretion of extracellular proteolytic enzymes.

### 3.5. AoMbp1 Is Required for Stress Resistance and Nutrient Utilization

**Figure 5:**
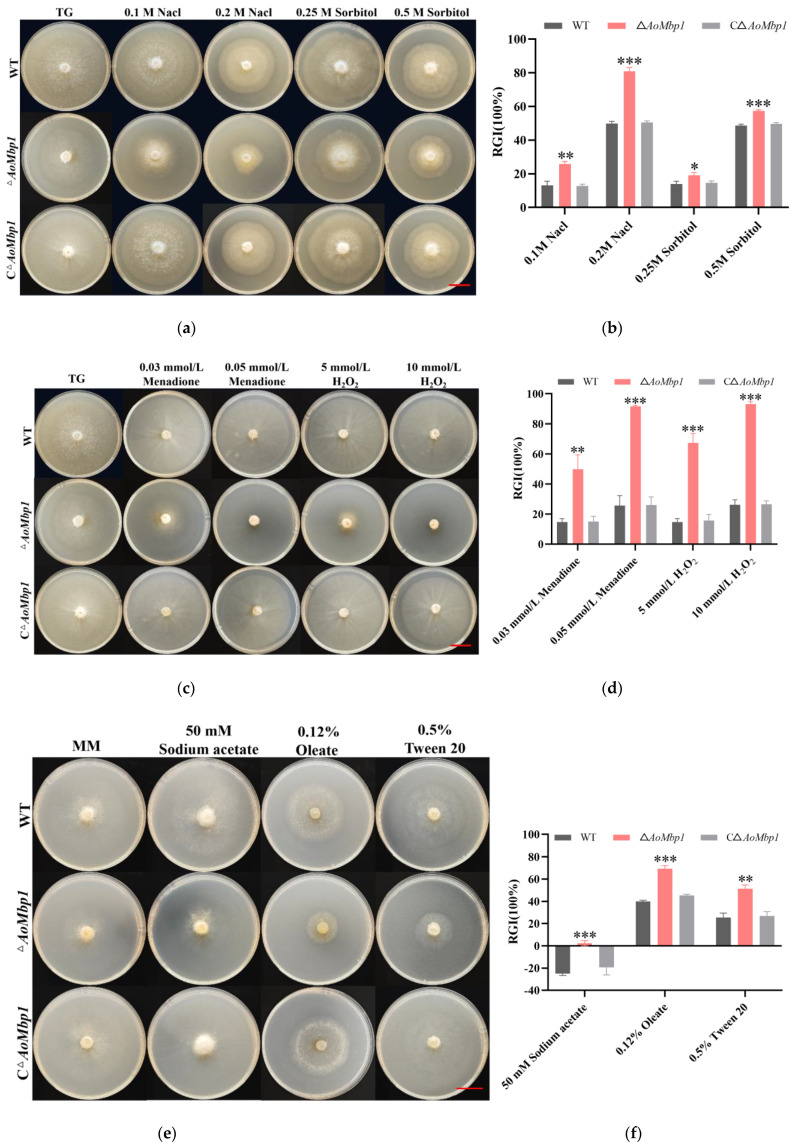

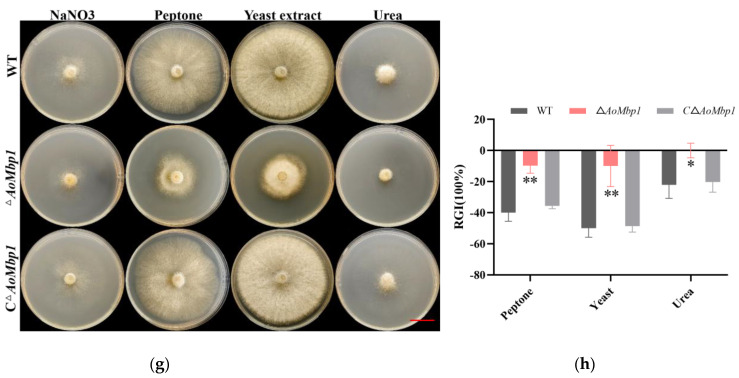
The Δ*AoMbp1* strain displays increased sensitivity to hyperosmotic, oxidative, and nutrient stresses. (**a**) Colony morphology of WT, Δ*AoMbp1*, and CΔ*AoMbp1* strains on TG agar supplemented with NaCl (0.1, 0.2 M) or sorbitol (0.25, 0.5 M) after 5 days at 28 °C. Scale bar, 2 cm. (**b**) RGI of strains under hyperosmotic stress. Data are mean ± SD (*n* = 3 biological replicates). * *p* < 0.05, ** *p* < 0.01, *** *p* < 0.001 vs. WT (Tukey’s HSD test). (**c**) Colony morphology on TG agar containing H_2_O_2_ (5, 10 mM) or menadione (0.03, 0.05 mM). Scale bar, 2 cm. (**d**) RGI of strains under oxidative stress. Data are mean ± SD (*n* = 3). ** *p* < 0.01, *** *p* < 0.001 vs. WT. (**e**) Colony morphology on minimal medium (MM) with different fatty acid sources. Scale bar, 2 cm. (**f**) RGI of strains under fatty acid stress. Data are mean ± SD (*n* = 3). ** *p* < 0.01, *** *p* < 0.001 vs. WT. (**g**) Colony morphology on MM with different nitrogen sources. Scale bar, 2 cm. (**h**) RGI of strains grown on different nitrogen sources. Data are mean ± SD (*n* = 3). * *p* < 0.05, ** *p* < 0.01 vs. WT.

Under both hyperosmotic and oxidative stress conditions, all tested strains exhibited varying degrees of growth inhibition. The Δ*AoMbp1* strain demonstrated significantly enhanced sensitivity to these stresses compared to the WT and CΔ*AoMbp1* strains. Under hyperosmotic stress conditions, the Δ*AoMbp1* mutant showed markedly enhanced growth inhibition (Figure 5a,b) with significantly higher Relative Growth Inhibition (RGI) values (Figure 5b). For instance, the Δ*AoMbp1* strain exhibited a 31.02% increase in RGI value under 0.2 M NaCl stress compared to the WT strain. Similarly, the mutant strain displayed pronounced sensitivity to oxidative stress agents, demonstrating substantially elevated RGI values relative to control strains (Figure 5c,d). The Δ*AoMbp1* strain showed RGI value increases of 65.87% with 0.05 mmol/L menadione and 66.80% with 10 mmol/L H_2_O_2_ compared to the WT strain. When cultured on media containing sodium acetate, Tween, or oleic acid as carbon sources, the mutant exhibited stronger growth suppression and increased RGI values compared to both WT and complemented strains (Figure 5e,f). The Δ*AoMbp1* strain demonstrated a 29.51% increase in RGI value with 0.12% oleate. Under different nitrogen source conditions, the Δ*AoMbp1* strain showed significantly elevated RGI values (Figure 5g,h), including a 40.04% increase with yeast extract. These findings demonstrate that deletion of *AoMbp1* substantially impairs the ability of *A. oligospora* to tolerate both hyperosmotic and oxidative stresses while reducing its capacity to utilize various fatty acids and nitrogen sources.

### 3.6. Transcriptome Analysis of the WT and ΔAoMbp1 Strains

**Figure 6:**
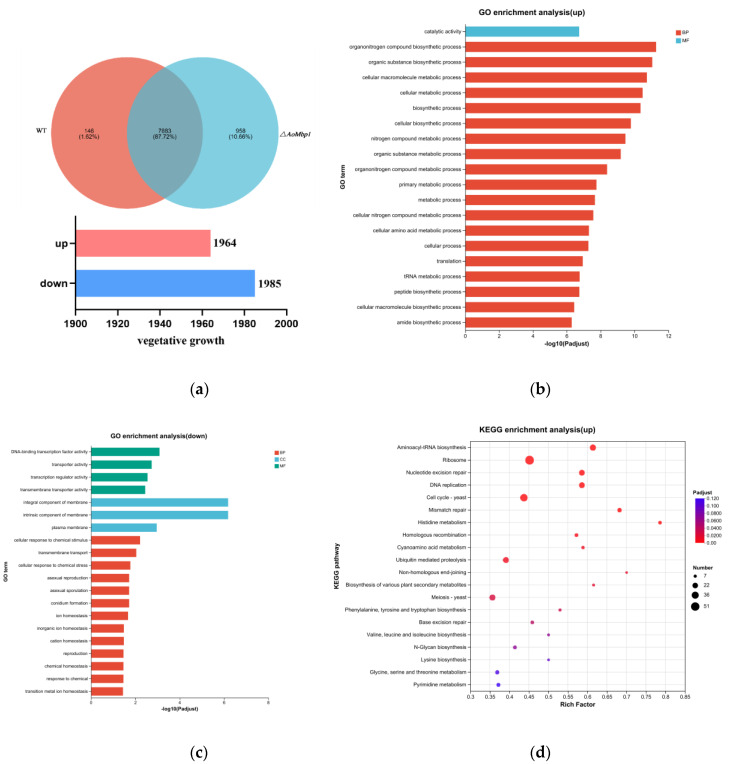

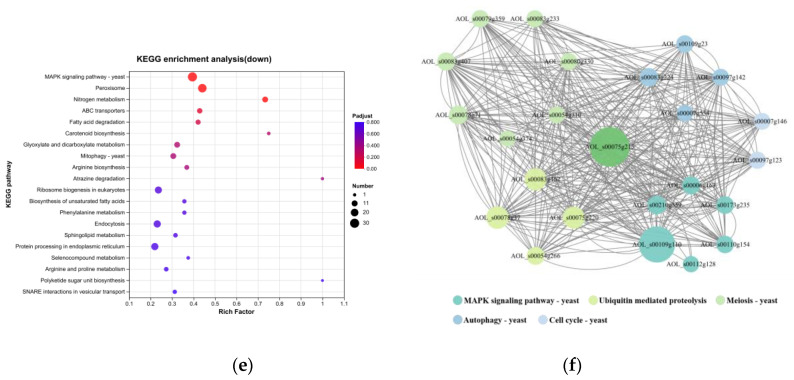
Transcriptomic analysis of the WT and Δ*AoMbp1* strains. (**a**) Statistics of differentially expressed genes (DEGs) in the Δ*AoMbp1* strain compared to the WT strain. (**b**,**c**) Gene Ontology (GO) enrichment analysis of DEGs. (**d**,**e**) KEGG pathway enrichment analysis of DEGs. (**f**) Prediction of potential *AoMbp1*-interacting proteins based on the STRING database and transcriptomic data.

The quality of the transcriptome sequencing data was evaluated (Figure S7a,b). The analysis revealed that 87.23–90.11% of the sequenced reads were successfully aligned to the *A. oligospora* reference genome. The error rate across all six samples was less than 0.02%, while the Q20 and Q30 quality scores ranged from 95.00% to 95.35%, and the GC content varied between 46.99% and 47.56%. These metrics indicate that the sequencing data were of high quality (Figure S7c). RT–qPCR validation of five DEGs confirmed the reliability of the RNA-seq data. The expression levels of four randomly selected genes (AOL_s00006g570, AOL_s00210g122, AOL_s00054g525, AOL_s00054g29) and *AoSho1* (AOL_s00078g396) were all consistent with the transcriptomic results (Figure S7d). Compared with the WT strain, the Δ*AoMbp1* mutant exhibited 1964 significantly upregulated and 1985 downregulated genes (Figure 6a and Table S2).

Gene Ontology (GO) enrichment analysis showed that upregulated genes were primarily associated with biosynthetic processes, including those for organonitrogen compound biosynthetic process, organic substance biosynthetic process, cellular macromolecule metabolic process, cellular metabolic process, catalytic activity, among others (Figure 6b). In contrast, downregulated genes were significantly enriched in processes such as cellular responses to chemical stimulus, transmembrane transport, cellular response to chemical stress, asexual reproduction, asexual spore sporulation, conidium formation, integral component of membrane, intrinsic component of membrane, plasma membrane, DNA-binding transcription factor activity, transport activity, transcriptional regulator activity, transmembrane transporter activity, among others (Figure 6c).

KEGG pathway enrichment analysis revealed that the upregulated DEGs were significantly enriched in pathways such as aminoacyl-tRNA biosynthesis, ribosome, nucleotide excision repair, DNA replication, the cell cycle (yeast), mismatch repair, and histidine metabolism (Figure 6d). In comparison, the downregulated DEGs were significantly enriched in pathways such as the mitogen-activated protein kinase (MAPK) signaling pathway, peroxisome pathway, and nitrogen metabolism pathway (Figure 6e).

Using the STRING database, a protein interaction network was constructed to identify proteins potentially functionally related to or directly interacting with *AoMbp1*. These results suggest that *AoMbp1* may play a regulatory role in several key cellular processes, such as the MAPK signaling pathway, ubiquitin-mediated protein hydrolysis, meiosis, autophagy, and cell cycle regulation (Figure 6f). Notably, combined transcriptome analysis revealed several specific proteins, including the mitogen-activated protein kinase *Hog1* (AOL_s00109g23), which is linked to the MAPK pathway and mitophagy; the cyclin-dependent kinase Cdc28 (AOL_s00210g359), associated with meiosis, the cell cycle, and the MAPK pathway; and the serine/threonine protein kinase Cdc5 (AOL_s00078g71), involved in cell cycle regulation and meiosis. Additionally, the regulatory subunit B of protein phosphatase PP2A (AOL_s00007g146) was notable for its role in the cell cycle.

### 3.7. Functional Analysis of DEGs

**Figure 7:**
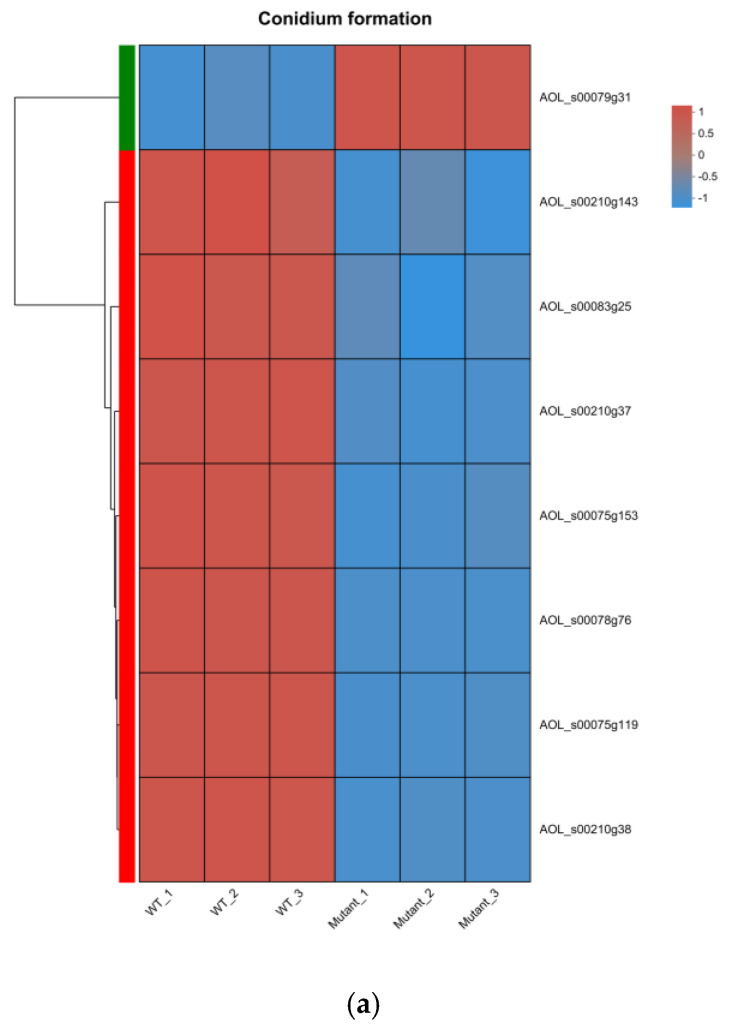

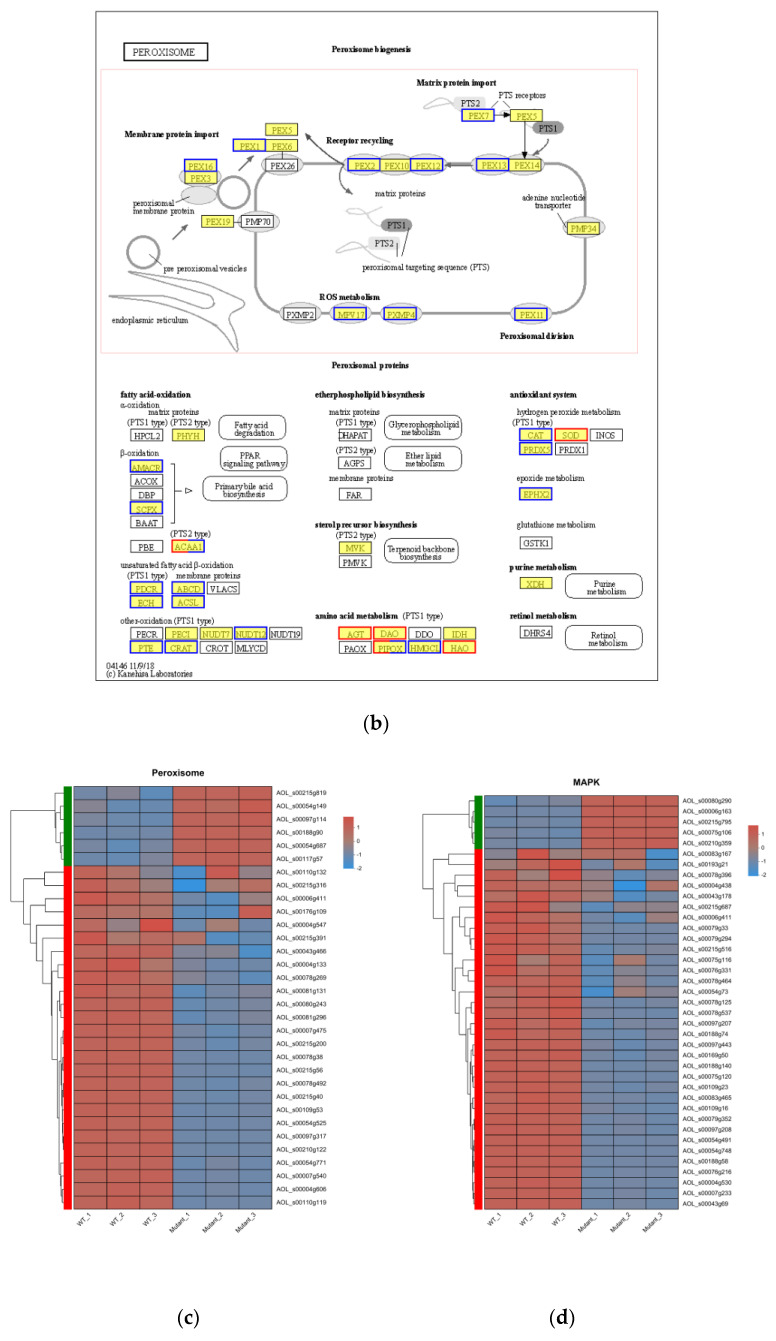

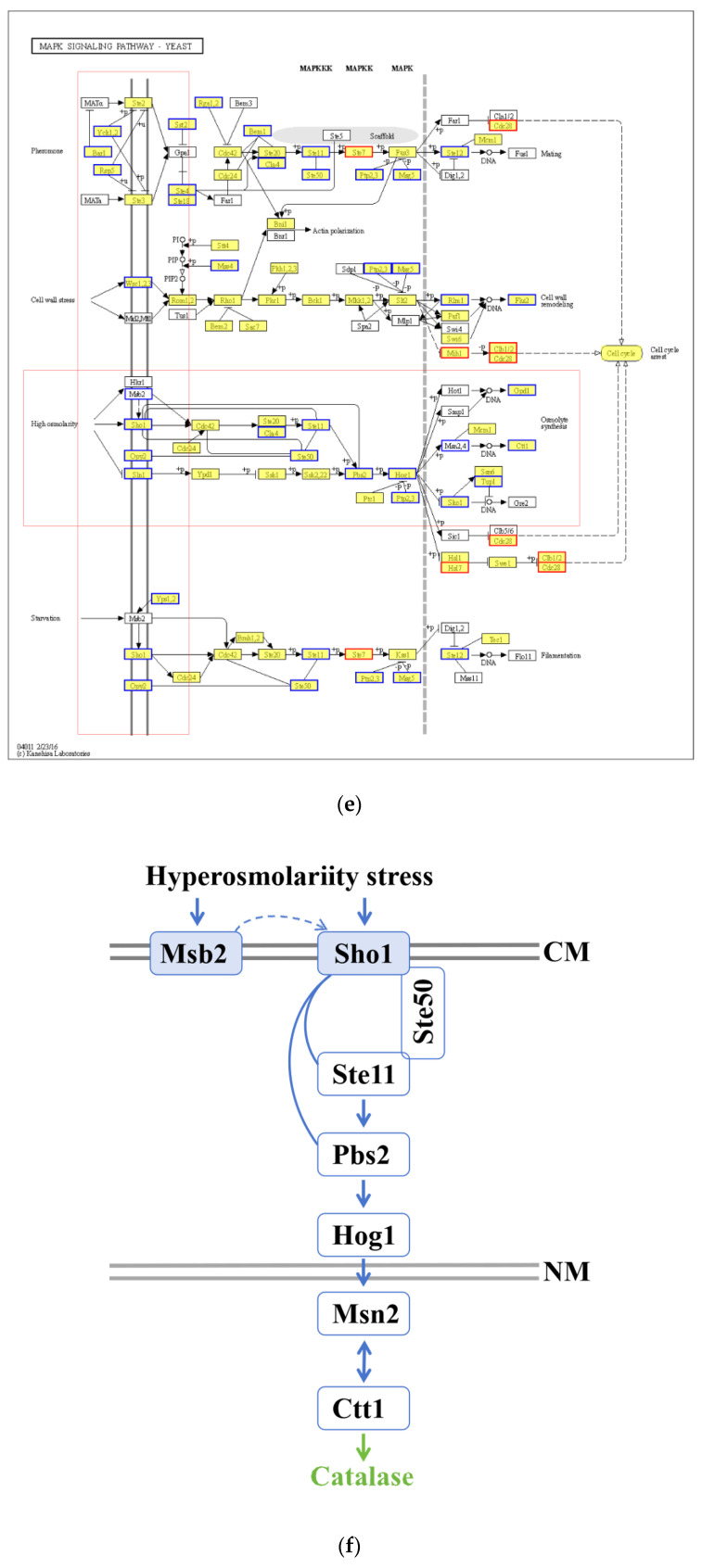

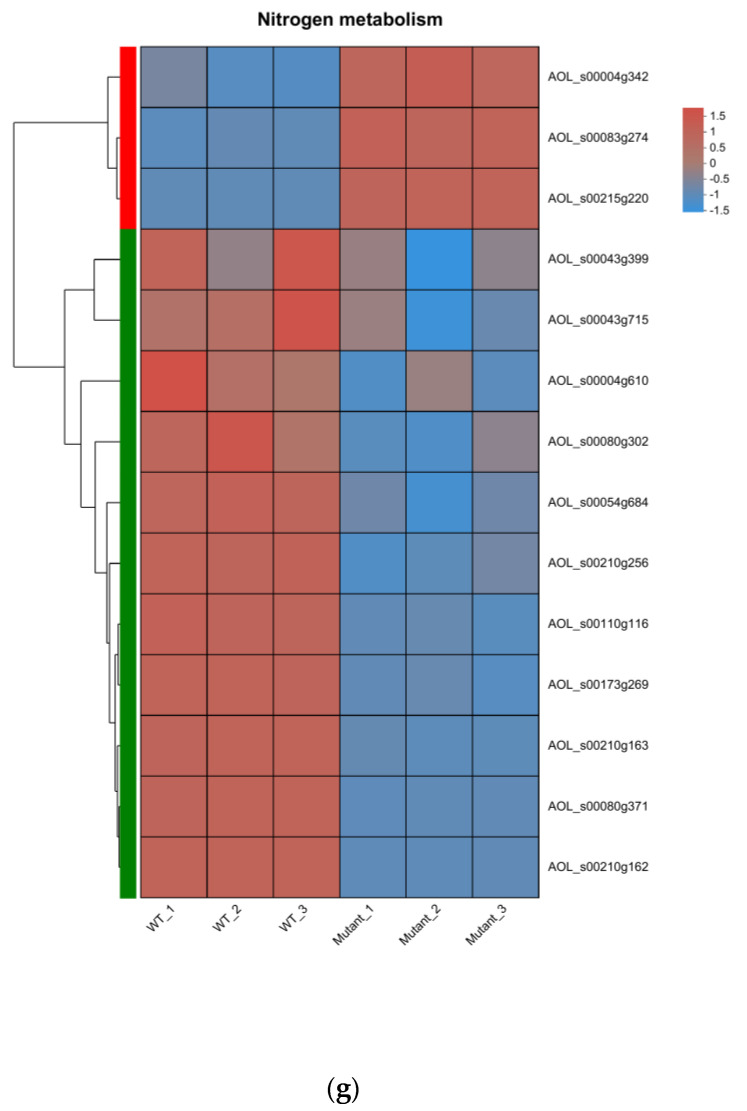
Analysis of differentially expressed genes (DEGs) involved in conidiation, peroxisomes, MAPK signaling, and nitrogen metabolism. (**a**) Heatmap of DEGs associated with conidiation. (**b**) Heatmap of DEGs related to peroxisomes. (**c**) Schematic diagram of the peroxisome pathway. (**d**) Heatmap of DEGs involved in MAPK signaling pathway. (**e**) Schematic of the MAPK signaling pathway. (**f**) Diagram of the Hog1-MAPK signaling branch. CM, cell membrane; NM, nuclear membrane. Double arrows indicate interacting proteins; green arrows represent pathways involved in gene regulation. Upregulated and downregulated genes are labeled in red and blue, respectively. (**g**) Heatmap of DEGs associated with nitrogen metabolism.

On the basis of Gene Ontology (GO) analysis, we performed a clustered heatmap analysis of DEGs related to conidium formation (GO: 0048315). The only significantly upregulated gene related to conidium formation in Δ*AoMbp1* was Chitin synthase (*Chs*, AOL_s00079g31), whereas the significantly downregulated genes included six *Chs* (AOL_s00210g38, AOL_s00078g76, AOL_s00210g143, AOL_s00210g37, AOL_s00075g119, AOL_s00075g153) and *StuA* (AOL_s00083g25) (Figure 7a). These results suggest that *AoMbp1* may play a crucial regulatory role in the sporulation process of *A. oligospora* by coordinately regulating multiple chitin synthases (*Chs*).

On the basis of KEGG analysis, we performed a clustered heatmap analysis of DEGs related to peroxisomes (pathway ID: map04146), the MAPK signaling pathway (pathway ID: map04011), and nitrogen metabolism (pathway ID: map00910). Among the DEGs involved in peroxisome synthesis (e.g., *Pex* and *Pxmp4*), those genes were significantly lower in the Δ*AoMbp1* strain than in the WT strain (Figure 7b). In addition, the expression of antioxidant enzyme-encoding genes involved in the response to oxidative stress (e.g., *Cat*,* Prdx5*) and genes involved in fatty acid β-oxidation (e.g., AMP-dependent synthase/ligase) were generally downregulated, whereas the expression of the superoxide dismutase gene *Sod2* was upregulated (Figure 7b,c), suggesting that *AoMbp1* is essential for maintaining normal peroxisome biogenesis, cellular redox homeostasis and fatty acid metabolism.

The Hog1-MAPK pathway, from the upstream sensor *Sho1* to the core kinase cascade (*Ste11*, *Pbs2*, *Hog1*) and then to the downstream transcription factor (*Msn2*) and effector gene (*Ctt1*), was significantly downregulated (Figure 7d–f). In addition, other key genes in the MAPK pathway, such as *Ste12* and *Ime2*, were also significantly downregulated. Notably, on the basis of the previous GO analysis results, genes whose expression was downregulated in the Δ*AoMbp1* deletion strain were significantly enriched in membrane components. Further analysis revealed that multiple genes encoding membrane proteins related to the MAPK pathway (such as *Sho1*, *Msb2*, and Wsc) were also significantly downregulated (Figure 7d,e), suggesting that *AoMbp1* may regulate the membrane protein-mediated MAPK signaling pathway. *AoSho1* (AOL_s00078g396), encoding a membrane protein, was significantly downregulated. Moreover, *AoSho1* serves as an upstream sensor of the Hog1-MAPK pathway, and its homologs in yeast may be regulated by Mbp1 [24]. Therefore, *AoSho1* was selected as a representative target gene for subsequent verification of *AoMbp1* transcriptional regulation.

Furthermore, the expression of those DEGs related to nitrogen metabolism was significantly downregulated in the Δ*AoMbp1* deletion strain (Figure 7g). These downregulated genes encoded key enzymes involved in nitrogen assimilation, transport, and catabolism, such as carbonic anhydrase (AOL_s00210g256), nitrate reductase (AOL_s00210g162), glutamate dehydrogenase (AOL_s00080g371), nitrite transporter (AOL_s00004g610), formamidase (AOL_s00043g399), and glutamate synthase (AOL_s00173g269).

### 3.8. AoMbp1 Directly Binds to the Promoter of the AoSho1 Gene

**Figure 8:**
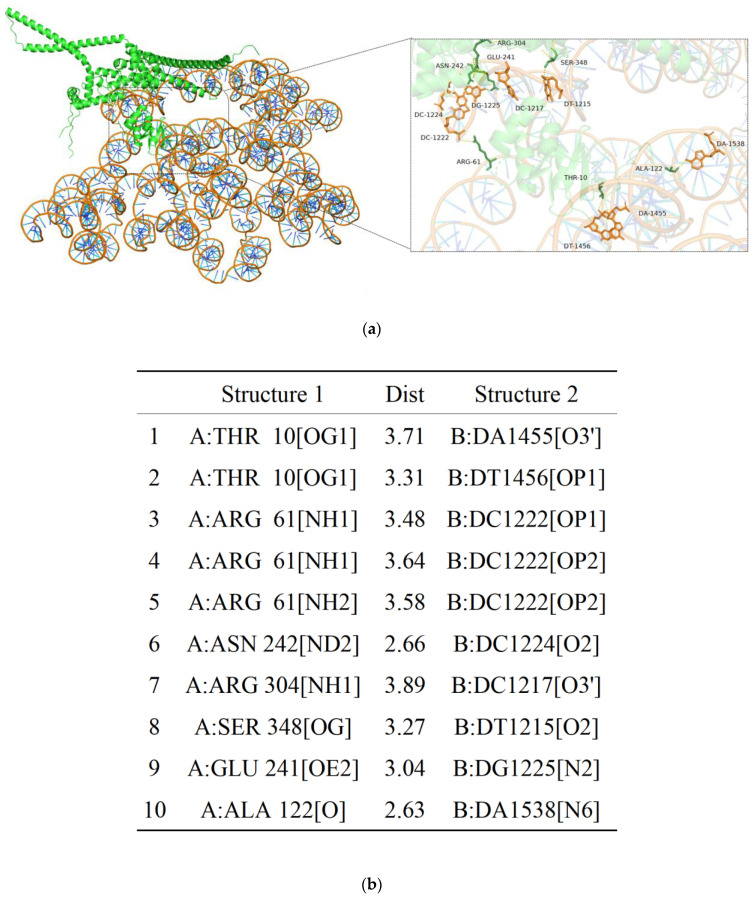

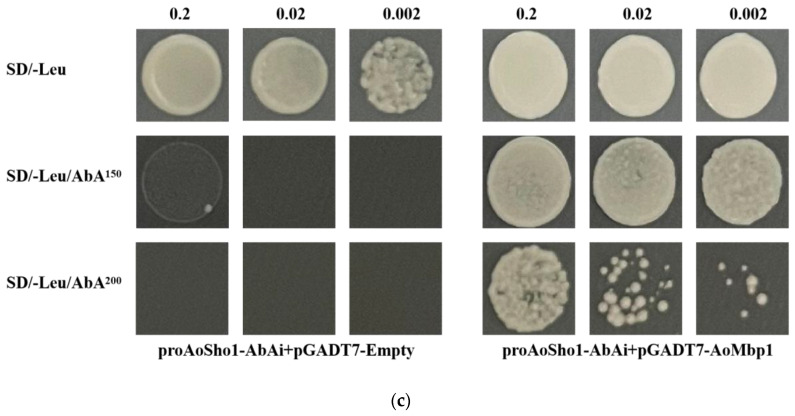
Verification of the interaction between *AoMbp1* and the promoter of *AoSho1*. (**a**,**b**) Molecular docking of the *AoMbp1* protein with the promoter of the *AoSho1* gene. A confidence score ≥ 0.7 indicates a high probability of binding; a score between 0.5 and 0.7 suggests potential binding; and a score < 0.5 indicates no binding. Visualization of hydrogen bonds at the protein–DNA binding interface. (**c**) Yeast one-hybrid assay evaluating the interaction between *AoMbp1* and the promoter of the *AoSho1* gene.

**Figure 9:**
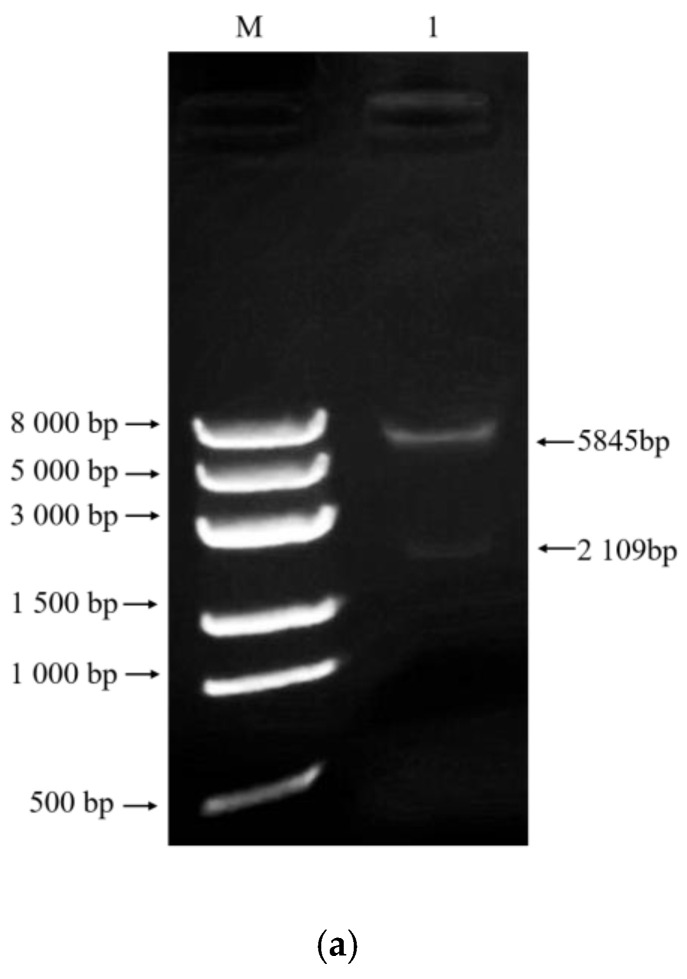

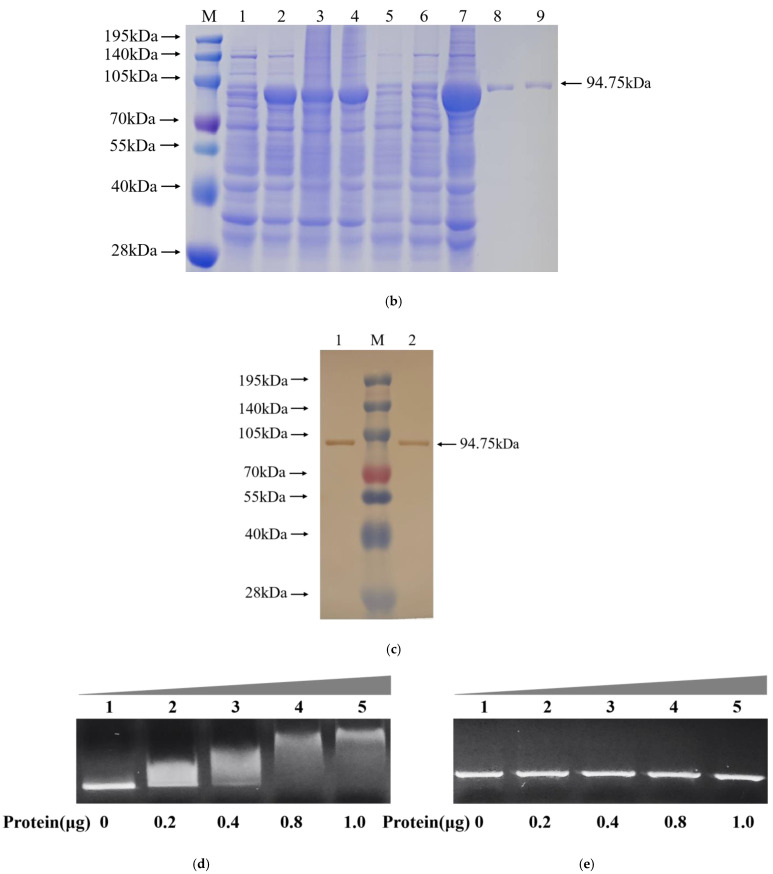
EMSA analysis of the interaction between *AoMbp1* and the *AoSho1* promoter. (**a**) Identification of recombinant plasmid pET-32a-*AoMbp1* by double enzyme digestion. M, DNA marker; lane 1, double enzyme digest of pET-32a-*AoMbp1*. (**b**) Expression analysis of the recombinant *AoMbp1* protein. M, prestained protein marker; lane 1, pET-32a vector induced for 6 h; lanes 2–4, precipitate of induced recombinant protein; lane 5, uninduced control; lane 6, supernatant after cell lysis; lane 7, precipitate after lysis; lanes 8–9, purified *AoMbp1* protein. (**c**) Western blot analysis of the recombinant *AoMbp1* protein. (**d**) EMSA showing the binding of purified *AoMbp1* protein to the *AoSho1* promoter. (**e**) Negative control showing no binding of BSA to the *AoSho1* promoter.

To experimentally verify the interaction between *AoMbp1* and its target promoter, we performed molecular docking, Y1H, and EMSA. Although no typical MluI box (5′-ACGCGT-3′) was detected in the *AoSho1* promoter region, a similar sequence (5′-CGCG-3′) was identified (Figure S8a,b). Molecular docking revealed strong binding affinity between *AoMbp1* and the 1000bp *AoSho1* promoter region, with a confidence score of 0.9456. Visualization using PDBePISA and PyMOL indicated the presence of 10 potential hydrogen bonds at the binding interface (Figure 8a,b). In the Y1H assay, yeast strains co-transformed with pGADT7-*AoMbp1* and the proAoSho1-AbAi bait vector grew vigorously on SD/-Leu medium containing 150 or 200 ng/mL aureobasidin A (AbA), whereas negative controls showed no growth (Figure 8c), indicating a specific interaction. EMSA results demonstrated a dose-dependent mobility shift in the *AoSho1* promoter fragment upon incubation with increasing concentrations of purified *AoMbp1* protein (Figure 9a–d). No shift was observed with bovine serum albumin (BSA) as a nonspecific control (Figure 9e), confirming in vitro binding specificity.

## 4. Discussions

**Figure 10:**
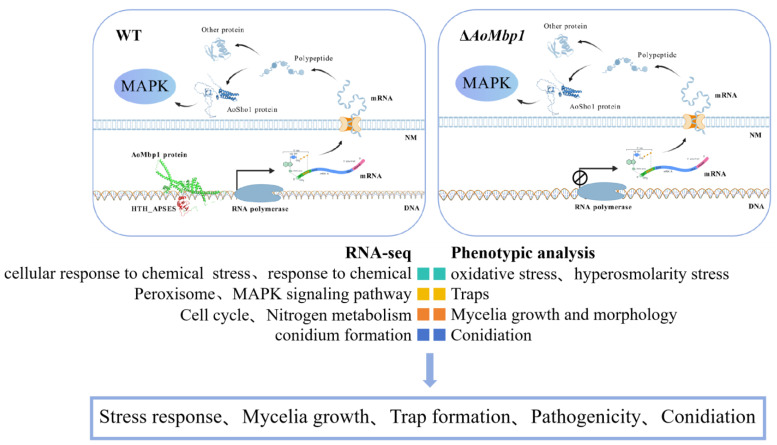
A proposed model for *AoMbp1* in regulating development and stress responses in *A. oligospora*. The APSES transcription factor *AoMbp1* modulates the expression of downstream genes (including *AoSho1*), thereby influencing the Hog1-MAPK signaling pathway. *AoMbp1* is critical for conidiation, trap formation, hyphal growth, and adaptation to oxidative and osmotic stresses.

Taken together, our analyses demonstrate that deletion of *AoMbp1* leads to severe phenotypic defects, including restricted mycelial growth, abnormal hyphal morphology, significantly impaired conidiation with aberrant conidial morphology, markedly reduced trap formation, and enhanced sensitivity to osmotic and oxidative stresses. Transcriptome profiling further validated these phenotypic observations, collectively confirming the essential role of *AoMbp1* in regulating hyphal development, conidiation, stress adaptation, and pathogenicity in *A. oligospora* (Figure 10).

Building on the conserved role of Mbp1 as a key regulator of the G1/S transition in yeast, we investigated its function in hyphal morphology and cell cycle regulation in *A. oligospora*. The Δ*AoMbp1* mutant exhibited swollen hyphae, increased septation, and a reduced growth rate, suggesting dysregulation of cell cycle progression, a process known to be essential for septation and hyphal elongation [25]. Transcriptomic analysis revealed significant upregulation of genes involved in fundamental cell cycle-related pathways, including aminoacyl-tRNA biosynthesis, ribosome biogenesis, nucleotide excision repair, DNA replication, cell cycle, and mismatch repair (Figure 6d). Notably, expression of the DNA damage response gene *Rnr1* (AOL_s00110g137) increased by 2.48-fold. In yeast, Mbp1 directly binds the *Rnr1* promoter to regulate its transcription. These results suggest that *AoMbp1* deletion disrupts the G1/S transition and induces DNA replication stress, leading to genomic instability and activation of DNA damage response mechanisms. This diversion of cellular resources toward repair processes likely impairs normal hyphal elongation and coordinated cell division, resulting in aberrant morphology and growth delay. Additionally, these disruptions may indirectly affect conidiation, which requires precise cell cycle control.

In contrast, downregulated genes were enriched in pathways associated with cellular stress responses, such as the MAPK signaling pathway (Figure 6e). The Δ*AoMbp1* strain exhibited heightened sensitivity to hyperosmotic stress, correlated with broad downregulation of the Hog1-MAPK signaling pathway (Figure 7f). In *A. oligospora*, *Hog1* acts as a core kinase of the HOG-MAPK pathway, regulating hyperosmotic stress responses, trap morphogenesis, and hyphal development [21,26]. Impaired *Hog1* signaling, a central coordinator of osmotic stress responses in fungi, likely underlies this severe sensitivity. Sho1, a conserved membrane sensor, activates the MAPK cascade via the *Sho1*-*Ste11*-*Pbs2*-*Hog1* pathway and is critical for fungal development, pathogenicity, and responses to diverse stresses [27,28,29,30]. Our Y1H and EMSA experiments confirmed direct binding of *AoMbp1* to the *AoSho1* promoter in vitro and in yeast systems. However, it must be noted that the absence of in vivo validation through chromatin immunoprecipitation (ChIP) represents a key limitation of this study. While our data provide strong evidence for this interaction and support a model where *AoMbp1* modulates the osmotic stress response by transcriptionally regulating *AoSho1*, definitive confirmation under native chromatin conditions awaits future investigation.

*AoMbp1* is also implicated in oxidative stress response and metabolic regulation, processes crucial for environmental adaptation and trap development. The Δ*AoMbp1* mutant showed increased sensitivity to oxidants and impaired growth under nutrient stress. Transcriptome analysis revealed significant downregulation of genes related to Peroxisome and nitrogen metabolism. Expression of the downstream transcription factor AoMsn2, which regulates oxidative stress responses and lipid metabolism, was also reduced [31]. In yeast, Pex34 interacts with Pex11, Pex25, and Pex2 to regulate peroxisome proliferation [32]. Similarly, in *A. oligospora*, Pex1 and Pex6 are associated with hyphal growth and trap formation [20], while Pex14/17 influences development and secondary metabolism [33]. Given the well-established roles of peroxisomes in β-oxidation and ROS detoxification, and their notable enrichment in trap structures, we hypothesize that the observed downregulation of peroxisome-related genes in Δ*AoMbp1* impairs peroxisomal function. This dysfunction likely reduces cellular ROS scavenging capacity and disrupts fatty acid metabolism, which collectively contributes to the growth deficiencies and increased oxidative stress sensitivity observed in the mutant.

Deletion of *AoMbp1* also markedly reduced conidial surface hydrophobicity, induced morphological abnormalities including defective septa, and significantly decreased spore production. Transcriptome analysis revealed that *AoMbp1* deletion significantly downregulated the expression of chitin synthase (*Chs*) genes and *RodA* (AOL_s00006g570). In *Aspergillus fumigatus*, *RodA* is a key hydrophobin critical for spore formation, surface permeability, hydrophobicity, and immune evasion [34]. Coordinated expression of *Chs* family members is essential for fungal morphology [35,36]. For instance, in *Aspergillus nidulans*, specific chitin synthases are required for septal chitin synthesis, a major structural component of fungal cell walls and septa [37]. Therefore, *AoMbp1* likely coordinates chitin synthesis and distribution through regulating *Chs* genes and RodA, thereby influencing conidial septum formation, morphology, and environmental adaptation.

The pleiotropic effects observed in the *AoMbp1* deletion mutant originate from the integrated impact of direct transcriptional regulation and indirect secondary consequences. Molecular characterization identified *AoSho1* as a direct transcriptional target, establishing a definitive mechanistic link for *AoMbp1*-mediated regulation of stress sensitivity and trap formation through the MAPK pathway. In contrast, the broader developmental and growth impairments appear to stem from secondary effects that manifest as functional disruptions across three distinct levels. Developmental processes including asexual reproduction, sporulation, and conidial formation show significant defects, while metabolic functions exhibit systematic dysregulation in organonitrogen compound biosynthesis, macromolecular metabolism, and transmembrane transport. Concurrently, stress response capabilities demonstrate substantially diminished capacity to counter chemical and environmental challenges. These functional deficiencies find molecular correlation through coordinated downregulation of core pathways including MAPK signaling, peroxisome function, and nitrogen metabolism. This transcriptional reprogramming is accompanied by broad suppression of key molecular functions, particularly transmembrane transporter activity and DNA-binding transcription factor activity. Furthermore, fundamental cellular processes involving aminoacyl-tRNA biosynthesis, ribosomal function, DNA replication, and repair mechanisms are significantly compromised.

Building upon these comprehensive findings, we propose an integrated “Offense-Defense-Metabolism” model wherein *AoMbp1* serves as a master transcriptional coordinator. During the ecological transition from saprophytism to predation, elevated *AoMbp1* expression orchestrates the coordinated regulation of genes involved in membrane composition, MAPK signaling, peroxisome biogenesis, fatty acid degradation, and nitrogen metabolism. Within the offense module, *A. oligospora* detects nematode-derived signals through membrane-localized GPCRs, transducing these signals to activate the intracellular MAPK pathway, with *AoMbp1* facilitating trap morphogenesis and promoting active infection through regulation of key signaling components. The defense module features *AoMbp1*-mediated enhancement of fungal tolerance to nematode-derived oxidative compounds via modulation of peroxisome biogenesis and associated functional genes, ensuring predation success through improved oxidative stress resistance. In the metabolism module, post-capture nutrient utilization is optimized through *AoMbp1* coordination of fatty acid β-oxidation and nitrogen metabolic pathways, enabling efficient energy allocation during predation. Collectively, our results demonstrate that the APSES transcription factor *AoMbp1* in *A. oligospora* has functionally diverged from its canonical cell cycle regulatory role in *S. cerevisiae*, representing an evolutionary adaptation to complex soil environments that enables the fungal transition from saprophytic to predatory existence through sophisticated integration of developmental, stress adaptation, and metabolic processes.

The pleiotropic role of *AoMbp1* in regulating conidiation, trap formation, and stress tolerance highlights its potential as a target for genetic strain improvement. This underscores the ability of master transcriptional regulators to reprogram fungal adaptation, as exemplified by the Zn_2_Cys_6_ transcription factor CtBOT6, whose overexpression activates the ABA-BOT gene cluster and converts the beneficial endophyte *Colletotrichum tofieldiae* into a potent pathogen [38]. Consequently, targeted modulation of *AoMbp1* expression, for example, through controlled overexpression, could generate *A. oligospora* strains with enhanced fitness under field conditions. Such strains are anticipated to exhibit improved conidial robustness, accelerated trap formation, and greater tolerance to environmental stresses common in agricultural soils. The identification of the *AoMbp1*–*AoSho1* regulatory axis provides a genetic framework and potential molecular markers, such as *AoSho1* expression levels, for tracking the performance of engineered strains. Thus, this work not only establishes a foundation for developing more effective biocontrol agents but also advances our understanding of how transcriptional circuits govern ecological strategies in fungi.

## 5. Conclusions

This study demonstrated that the APSES family protein *AoMbp1* is a global transcription factor that plays critical roles in regulating oxidative stress, hyperosmotic stress, pathogenicity, trap formation, mycelial growth, and conidial formation by modulating downstream target genes, such as *AoSho1*. These findings not only provide new insights into the regulatory mechanisms of the APSES family of transcription factors in *A. oligospora* but also lay a theoretical foundation for the development of genetically modified *A. oligospora*.

## Data Availability

The data will be made available upon request.

## Supplementary Materials

The following supporting information can be downloaded at https://www.mdpi.com/article/10.3390/jof11100736/s1. Figure S1: *AoMbp1* transcript levels are upregulated during conidiation and trap formation. Relative expression of *AoMbp1* in *Arthrobotrys oligospora* during conidiation (CON) and trap formation (TF) stages compared to the saprophytic stage, as determined by RT–qPCR. Gene expression was normalized to *β-tubulin*, with the saprophytic stage set as the calibrator (value = 1). Data are presented as mean ± SD (*n* = 3 biological replicates). Statistical significance was determined using one-way ANOVA with Dunnett's test against the saprophytic stage control (** *p* < 0.01, ** *p* < 0.001); Figure S2: Phylogenetic analysis of the APSES transcription factor family. (a) Phylogenetic tree of APSES proteins from different fungal species. Sc, *Saccharomyces cerevisiae*; Bb, *Beauveria bassiana*; Cf, *Ceratocystis fimbriata*; Ao, *A. oligospora*. (b) Summary of APSES transcription factor family members identified in *A. oligospora;* Figure S3: Analysis of the *AoMbp1* (*AOL_s00075g215*) promoter region in the *A. oligospora* strain. The relative positions of the *AOL_s00075g214* and *AOL_s00075g215* genes and the bidirectional promoter in the genome; Figure S4: Molecular characteristics of the *AoMbp1* protein of *A. oligospora.* Alignment analysis of *AoMbp1* amino acid sequences, with red highlighting indicating the highest conservation; Figure S5. Phylogenetic and conserved motif analyses based on the nucleotide sequence of the *AoMbp1* gene (neighbor-joining method). Figure S6. Construction and molecular validation of the *AoMbp1* deletion (Δ*AoMbp1*) and complementation (CΔ*AoMbp1*) strains. (a) Schematic diagram of the strategy used for *AoMbp1* gene deletion and complementation in the *A. oligospora* XJ-2 strain. The Δ*AoMbp1* mutant was generated by replacing the *AoMbp1* coding sequence with a hygromycin B resistance cassette (*hph*). The CΔ*AoMbp1* strain was constructed by reintroducing the full-length *AoMbp1* gene under the control of its native promoter into the Δ*AoMbp1* mutant. (b) PCR amplification of the deletion strain and complementation strain. M: 5K DNA marker; lanes 1–2, WT strain; lane 3, CΔ*AoMbp1* strain; lanes 4–5, The semi-transform strain. (c) Transcriptomic analysis confirming the knockout of *AoMbp1*. Expression levels of *AoMbp1* in the WT and Δ*AoMbp1* strains cultured in YPSSA medium at 28 °C for 6 days. Data are from RNA sequencing (*n* = 3 biological replicates). *** *p* < 0.001 (one-way ANOVA with Tukey's HSD test). (d) RT–qPCR validation of *AoMbp1* expression restoration in the complementation strain. Relative transcript levels of *AoMbp1* in the WT and CΔ*AoMbp1* strains after cultivation in YPSSA medium at 28 °C for 6 days. Data are presented as mean ± SD (*n* = 3). ns, not significant; Figure S7. Transcriptomic data quality assessment and validation. (a) Correlation heatmap of gene expression profiles among the six sequenced samples. (b) Principal component analysis (PCA) plot of the WT and Δ*AoMbp1* transcriptomes. (c) Summary statistics of RNA sequencing data. (d) RT–qPCR validation of RNA-seq results. Expression levels of five differentially expressed genes (DEGs) in the Δ*AoMbp1* strain relative to the WT are shown. Four randomly selected DEGs (*AOL_s00006g570*, *AOL_s00210g122*, *AOL_s00054g525*, *AOL_s00054g29*) and *AoSho1* (*AOL_s00078g396*) were analyzed. Gene expression was normalized to *β-tubulin*. Data are presented as mean ± SD (*n* = 3 biological replicates). ** *p* < 0.01, *** *p* < 0.001 (one-way ANOVA with Tukey's HSD test). Figure S8. Prediction of the interaction motif between the *AoMbp1* protein and the *AoSho1* promoter. (a) Sequence logo generated from the predicted binding site. (b) Potential DNA sequences within the *AoSho1* promoter identified as interaction targets for *AoMbp1*. Table S1. List of primers used in this study. Table S2. Up- and down-regulated differentially expressed genes (DEGs) in the *Arthrobotrys oligospora *Δ*AoMbp1* mutant versus the WT strain.
