# Supplementary material for: AoMbp1 Governs Conidiation and Trap Morphogenesis in Arthrobotrys oligospora Via Direct Transcriptional Activation of the MAPK Sensor AoSho1
Source: J Fungi (Basel). 2025 Oct 13;11(10):736. doi: 10.3390/jof11100736 (PMC12565038; doi:10.3390/jof11100736)
Supplement: Supplementary file 1 [file jof-11-00736-s001.zip › jof-3838071-supplementary.pdf]

Supplementary Materials

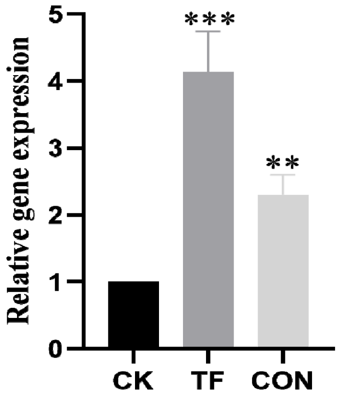

Fig. S1. *AoMbp1* transcript levels are upregulated during conidiation and trap formation.

Relative expression of *AoMbp1* in *Arthrobotrys oligospora* during conidiation (CON) and trap formation (TF) stages compared to the saprophytic stage, as determined by RT–qPCR. Gene expression was normalized to *β-tubulin*, with the saprophytic stage set as the calibrator (value = 1). Data are presented as mean ± SD (n = 3 biological replicates). Statistical significance was determined using one-way ANOVA with Dunnett's test against the saprophytic stage control (**P < 0.01, **P < 0.001).

a

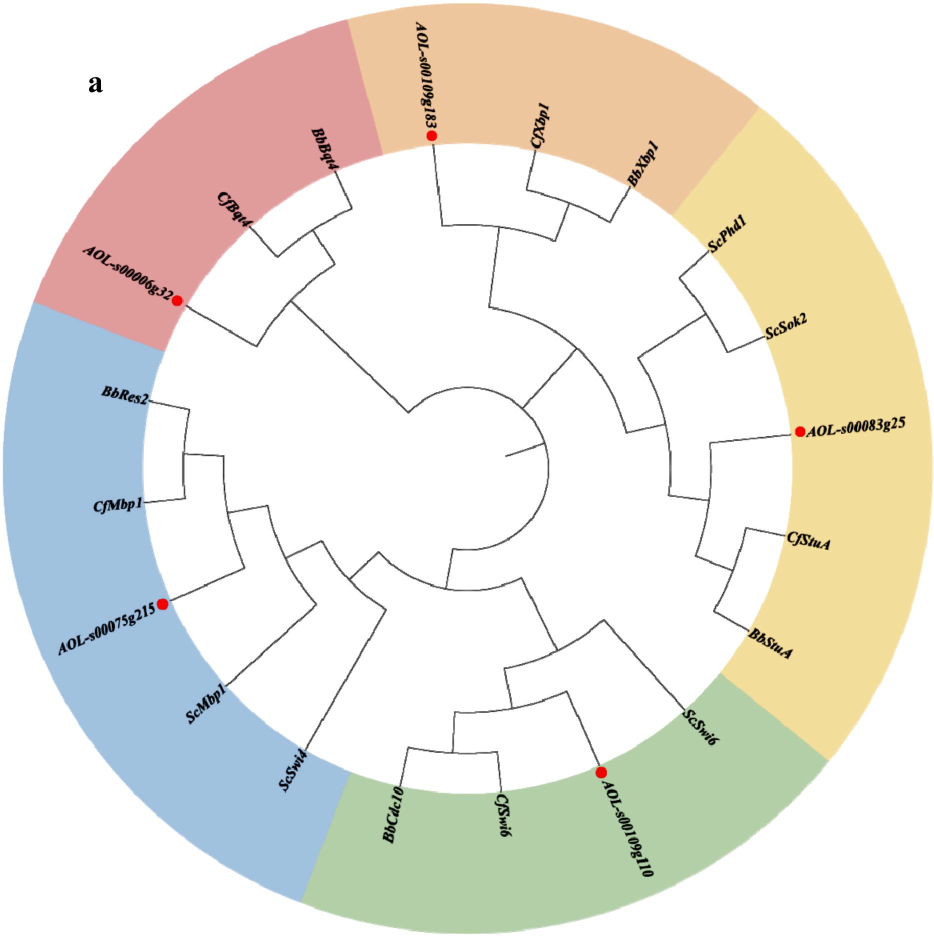

b

Accession	Gene	name
G1WZI1	AOL_s00006g32	AoBfp4
G1X8L6	AOL_s00075g215	AoMbp1
G1XG95	AOL_s00083g25	AoStuA
G1XK81	AOL_s00109g110	AoSwi6
G1XKF4	AOL_s00109g183	AoXbpl

Fig. S2. Phylogenetic analysis of the APSES transcription factor family.

(a) Phylogenetic tree of APSES proteins from different fungal species. Sc, *Saccharomyces cerevisiae*; Bb, *Beauveria bassiana*; Cf, *Ceratocystis fimbriata*; Ao, *A. oligospora*. (b) Summary of APSES transcription factor family members identified in *A. oligospora*.

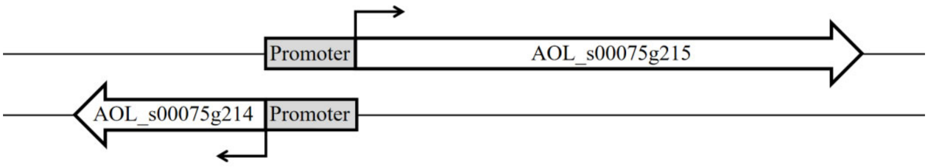

Fig. S3 Analysis of the AoMbp1(AOL_s00075g215) promoter region in the *A. oligospora* strain.

The relative positions of the AOL_s00075g214 and AOL_s00075g215 genes an d the bidirectional promoter in the genome.

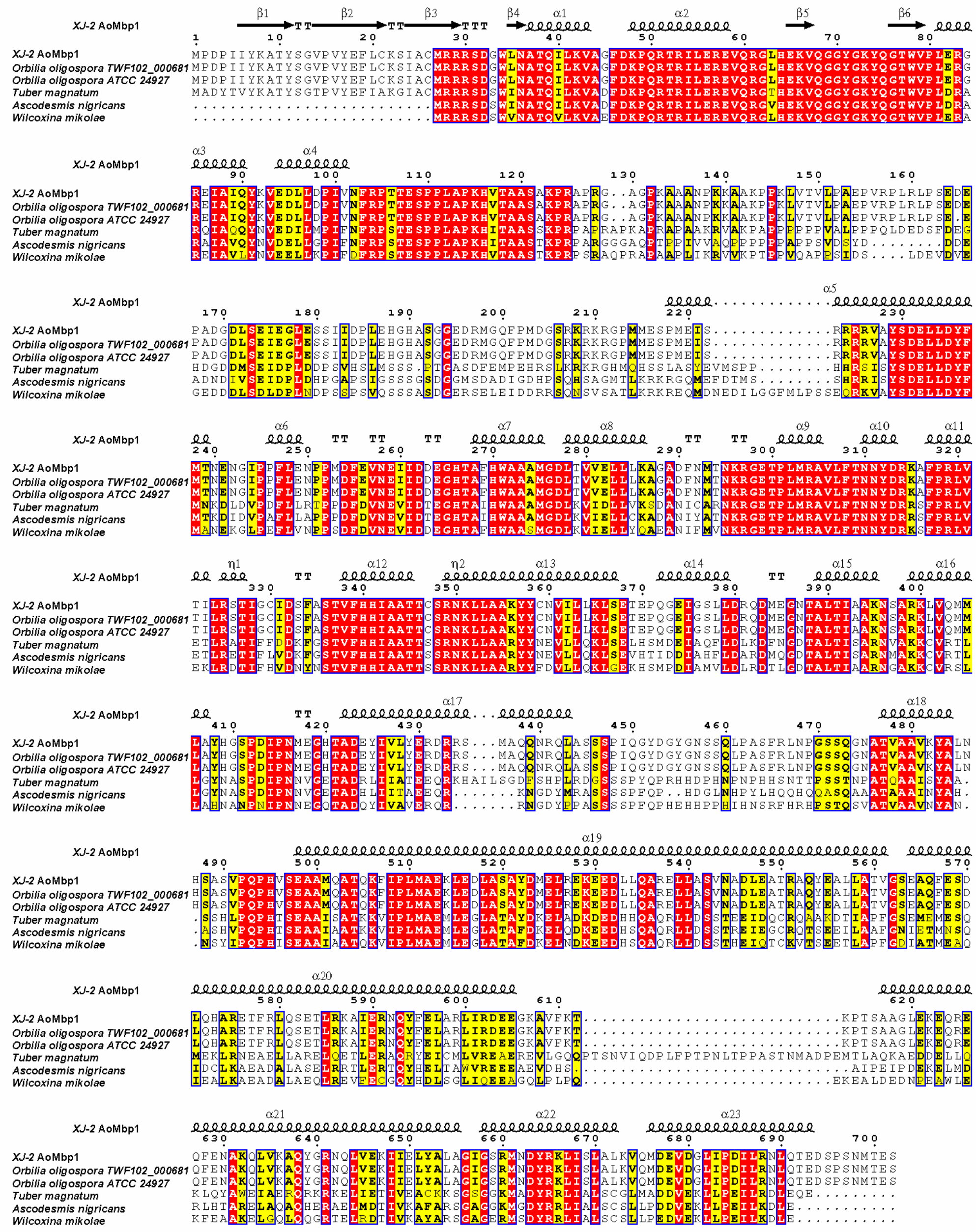

Fig. S4 Molecular characteristics of the AoMbp1 protein of *A. oligospora*. Alignment analysis of AoMbp1 amino acid sequences, with red highlighting in dicating the highest conservation.

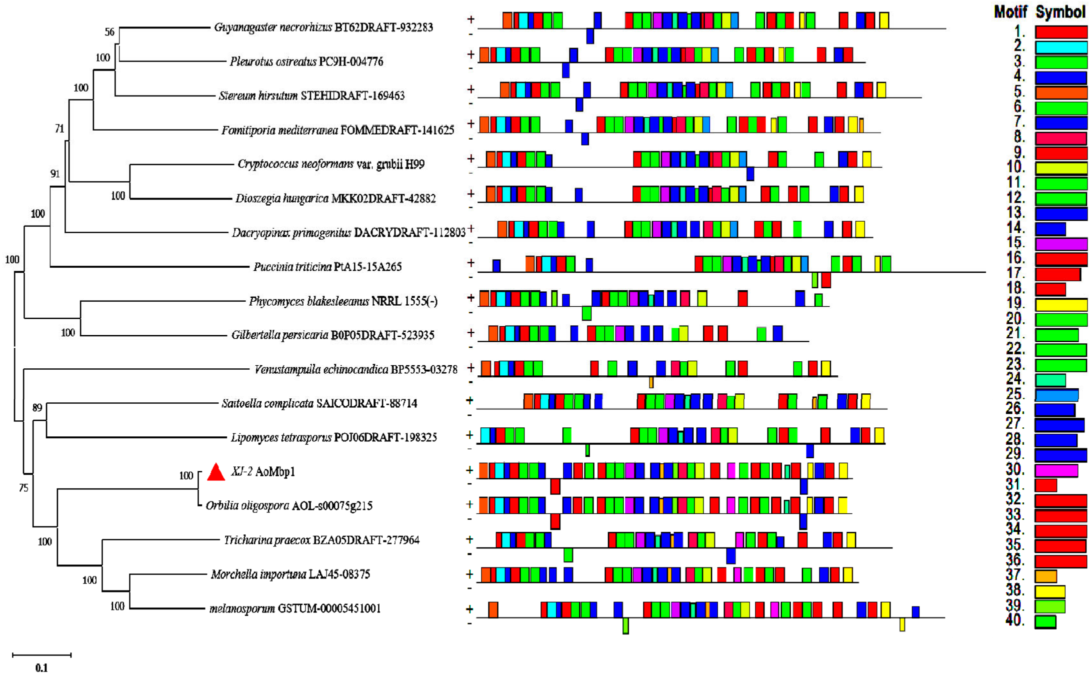

Fig. S5. Phylogenetic and conserved motif analyses based on the nucleotide sequence of the *AoMbp1* gene (neighbor-joining method).

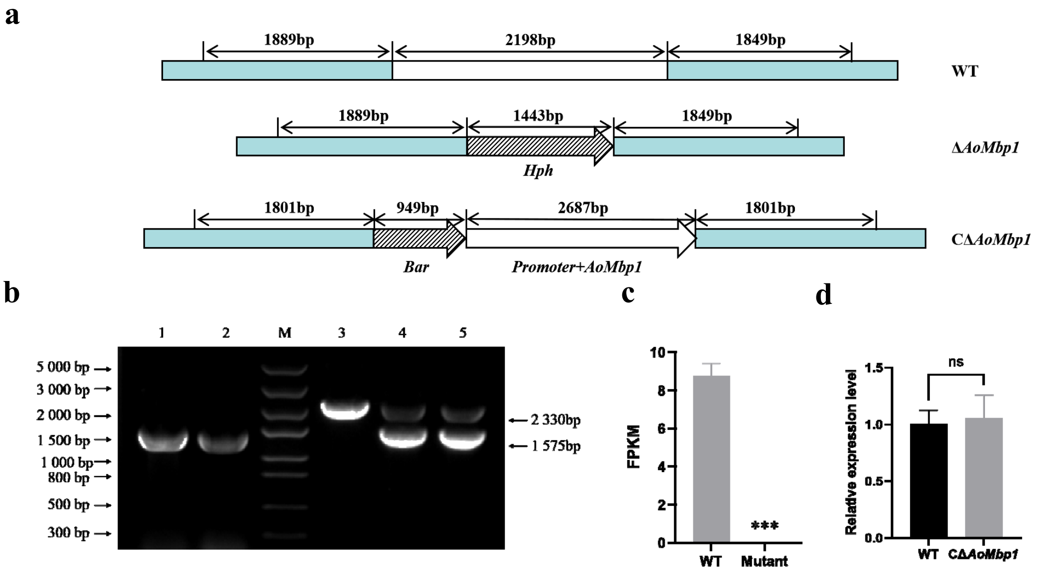

Fig. S6. Construction and molecular validation of the *AoMbp1* deletion (ΔAoMbp1) and complementation (CΔ*AoMbp1*) strains.

(a) Schematic diagram of the strategy used for *AoMbp1* gene deletion and complementation in the *A. oligospora* XJ-2 strain. The Δ*AoMbp1* mutant was generated by replacing the AoMbp1 coding sequence with a hygromycin B resistance cassette (hph). The CΔ*AoMbp1* strain was constructed by reintroducing the full-length AoMbp1 gene under the control of its native promoter into the Δ*AoMbp1* mutant. (b) PCR amplification of the deletion strain and complementation strain. M: 5K DNA marker; lanes 1–2, WT strain; lane 3, CΔ*AoMbp1* strain; lanes 4–5, The semi-transform strain. (c) Transcriptomic analysis confirming the knockout of AoMbp1. Expression levels of AoMbp1 in the WT and ΔAoMbp1 strains cultured in YPSSA medium at 28 °C for 6 days. Data are from RNA sequencing (n = 3 biological replicates). ***P < 0.001 (one-way ANOVA with Tukey's HSD test). (d) RT–qPCR validation of AoMbp1 expression restoration in the complementation strain. Relative transcript levels of AoMbp1 in the WT and CΔ*AoMbp1* strains after cultivation in YPSSA medium at 28 °C for 6 days. Data are presented as mean ± SD (n = 3). ns, not significant.

a

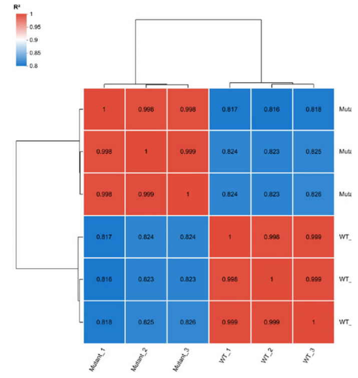

b

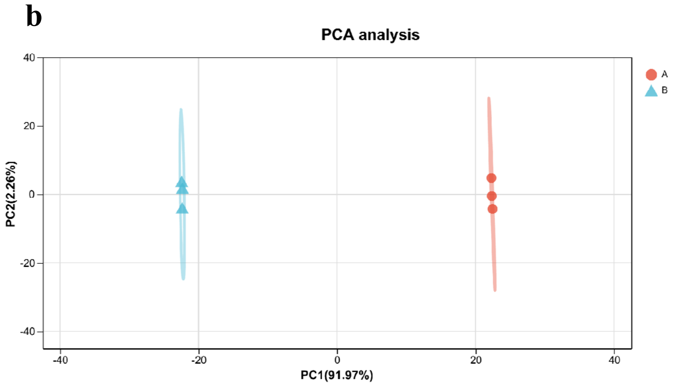

c

Sample	Raw reads	Raw bases	Clean reads	Clean bases	Error rate(%)	Q20(%)	Q30(%)	GC content(%)
WT_1	43739068	6604599268	43262730	6476476727	0.0125	98.42	95.12	47.13
WT_2	46128454	6965396554	45708810	6845401919	0.0125	98.41	95.08	46.99
WT_3	41874714	6323081814	41486106	6219028693	0.0124	98.47	95.28	47.04
Mutant_1	44826820	6768849820	44397572	6653947591	0.0124	98.5	95.35	47.56
Mutant_2	42480536	6414560936	42051960	6281616442	0.0126	98.39	95	47.6
Mutant_3	46171332	6971871132	45712060	6833761502	0.0125	98.43	95.15	47.56

d

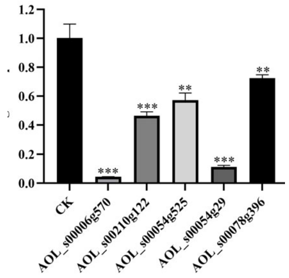

Fig. S7. Transcriptomic data quality assessment and validation.

(a) Correlation heatmap of gene expression profiles among the six sequenced samples. (b) Principal component analysis (PCA) plot of the WT and Δ*AoMbp1* transcriptomes. (c) Summary statistics of RNA sequencing data. (d) RT–qPCR validation of RNA-seq results. Expression levels of five differentially expressed genes (DEGs) in the Δ*AoMbp1* strain relative to the WT are shown. Four randomly selected DEGs (AOL_s00006g570, AOL_s00210g122, AOL_s00054g525, AOL_s00054g29) and AoSho1 (AOL_s00078g396) were analyzed. Gene expression was normalized to *β-tubulin*. Data are presented as mean ± SD (n = 3 biological replicates). **P < 0.01, ***P < 0.001 (one-way ANOVA with Tukey's HSD test).

a

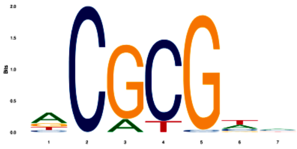

A [	55	0	15	0	0	35	33	]
C [	10	100	0	88	2	17	30	]
G [	18	0	85	0	98	9	18	]
T [	17	0	0	12	0	39	18	]

b

Promoter predictions for 1 eukaryotic sequence with score cutoff 0.80 (transcription start shown in larger font):

Promoter predictions for seq0 :

Start	End	Score	Promoter Sequence
69	119	0. 85	TAGGCTAAAATATCTAACCGGAGCTTTGGATCCTGGCGGCGGCGTGCAAT
309	359	0. 95	GGGAATGCGTGTATACGAGCGCGTATTGATTCTACTGTGCATTGAACCGT
464	514	0. 93	TAATGTAAAATAAAATAAATTCGGGGTCTCTCGCCCGCAGAGTCGACTAG
1009	1059	0. 86	CAGAATAATATATAATTACCAGCGGAGTGGTACATTTTGAAACGGGCCTT
1609	1659	0. 97	CTTTTCCCTCTTAAAAGCGACCAACCATCCTTCCACTCCACTCAACCAAA
1739	1789	1. 00	TAATAATTGGAAAAAAAAGCTCCCGGTCGAGCCATTAGTTATCATCGTTA

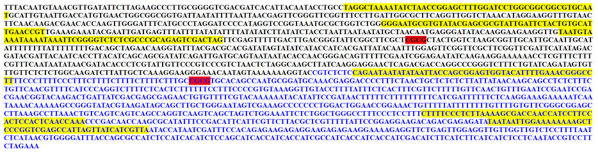

Fig. S8. Prediction of the interaction motif between the AoMbp1 protein and the *AoSho1* promoter.

(a) Sequence logo generated from the predicted binding site. (b) Potential DNA sequences within the *AoSho1* promoter identified as interaction targets for AoMbp1.

Table S1. List of primers used in this study

Primer name	Primer sequence （5’→3’）
P1	ATGCCGGATCCTATCATCTACAA
P2	TTAAGACTCTGTCATATTCGACGGA
P3	CTAATGGATTTGAATGGCTGCTC
P4	ATCCACTTAACGTTACTGAAATCTCCAACTTACACCACTATACGTCGCCTTGT
P5	CTCCTTCAATATCATCTTCTGTCTCCGACAATGGACGAAGTTGACGGGTT
P6	AGTCTTGAGGAACTCCCAATCGT
P7	ACAAGGCGACGTATAGTGGTGTAAGTTGGAGATTTCAGTAACGTTAAGTGGAT
P8	AACCCGTCAACTTCGTCCATTGTCGGAGACAGAAGATGATATTGAAGGAGC
PM1	GGATTTGAATGGCTGCTCTTCCACGT
PM2	CTTGAGGAACTCCCAATCGTAGTCGATG
proAoSho1-F	CTTGAATTCGAGCTCGGTACCTCTTATTGCTCAAAGGAAGGG
proAoSho1-R	GTCGACAGATCCCCGGGTACCTTTCTAGAAGGACGGTATTGAG
AoMbp1- F	CCATGGAGGCCAGTGAATTCATGCCGGATCCTATCATCTACA
AoMbp1-R	TGCCCACCCGGGTGGAATTCAGACTCTGTCATATTCGACGGAC
F1	*GG*GGTACCATGCCGGATCCTATCATCTACAA
R1	*CCC*AAGCTTTTAAGACTCTGTCATATTCGACGGA
F2	TCTTATTGCTCAAAGGAAGGG
R2	TTTCTAGAAGGACGGTATTG
